# Ferrihydrite Supported on Steel Slags as Catalyst for the Hydrogenation of Nitroarenes: A Virtuous Cycle of Wastes

**DOI:** 10.1002/gch2.202500201

**Published:** 2025-07-29

**Authors:** Francesca Derobertis, Maria M. Dell'Anna, Nicoletta Ditaranto, Luca Nodari, Stefania Liuzzi, Ernesto Mesto, Emanuela Schingaro, Cristina Leonelli, Cecilia Mortalò, Antonino Rizzuti, Carlo Porfido, Piero Mastrorilli

**Affiliations:** ^1^ Dipartimento di Ingegneria Civile, Ambientale, del Territorio, Edile e di Chimica DICATECh Politecnico di Bari via Orabona 4 Bari 70125 Italy; ^2^ Dipartimento di Chimica and CSGI – Bari Unit Università degli Studi di Bari Aldo Moro via Orabona 4 Bari 70125 Italy; ^3^ Institute of Condensed Matter Chemistry and Technologies for Energy (ICMATE‐CNR) National Research Council (CNR) C.so Stati Uniti 4 Padova 35127 Italy; ^4^ Dipartimento ARCOD Politecnico di Bari via Orabona 4 Bari 70125 Italy; ^5^ Dipartimento di Scienze della Terra e Geoambientali Università degli Studi di Bari Aldo Moro via Orabona 4 Bari 70125 Italy; ^6^ Dipartimento di Ingegneria “Enzo Ferrari” Università degli Studi di Modena e Reggio Emilia Via P. Vivarelli 10 Modena 41125 Italy; ^7^ Dipartimento delle Scienze del Suolo delle Piante e degli Alimenti Di.S.S.P.A. Università degli Studi di Bari Aldo Moro via Orabona 4 Bari 70125 Italy

**Keywords:** ferrihydrite, nitroarenes, steel slags, transfer hydrogenation, waste valorization

## Abstract

This study deals with the reduction reaction of nitroarenes using hydrazine monohydrate as the reducing agent and iron‐supported steel slag as a novel green heterogeneous catalyst. Steel slag is a byproduct of the steel industry, which, due to its alkalinity, can act as a reactive support that can trigger the formation of catalytically active iron oxides/hydroxides. A systematic study is conducted to evaluate the catalytic activity of steel slags modified with the following salts (or mixtures): FeSO_4_·7H_2_O, FeCl_3_·6H_2_O, and FeCl_2_·4H_2_O. The modified steel slags are characterized by X‐ray powder diffraction, Mössbauer spectroscopy, scanning electron microscopy, scanning transmission electron microscopy, energy dispersive X‐ray spectroscopy, nitrogen sorption analysis, and X‐ray photoelectron spectroscopy. All iron‐supporting steel slags demonstrate active behavior in the hydrogenation of nitrobenzene at 80 °C with the best results, in terms of activity, selectivity, and recyclability achieved with the catalyst prepared from FeCl_3_·6H_2_O (**Fe3**). The scalability of the reaction is confirmed by carrying out a test on 12.5 mmol of substrate. The superiority of **Fe3** compared with the other studied materials is ascribed to its morphology and the remarkably high surficial area. The iron species active in the **Fe3** catalyst are noncrystalline oxo–hydroxo species of Fe(III) (2L‐ferrihydrite).

## Graphical abstract

Highly alkaline steel slags coming from industrial sites in Apulia (Italy) have the property of transforming iron(II) or iron(III) salts into amorphous nanocrystalline ferrihydrite, which coats the surface of the steel slag particles and confers to them a remarkable catalytic activity in the transfer hydrogenation of nitroarenes to anilines.

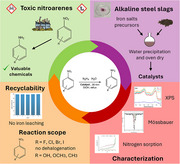

## 1 Introduction

Nitroarenes are a class of toxic, carcinogenic, and mutagenic compounds produced by human and industrial activities, primarily in the pharmaceutical and agricultural industries.^[^ ^1^ ^]^ The toxicity of these molecules is attributed to their high electron affinity, electronegativity, and reduction potential,^[^ ^1^ ^]^ which collectively drive the reduction to nitroarenes within the living organisms. This process may occur via the intermediacy of molecules such as nitrosobenzenes, hydroxylanilines, diazo, and azoxy compounds, contingent on the mechanism involved.^[^ ^2^, ^3^ ^]^ All aforementioned intermediates are toxic to humans.^[^ ^4^ ^]^ In particular, the initial stage of the reduction process involves the formation of a nitro radical anion via a reversible single‐electron transfer. This process is reversible due to the reaction with oxygen, which leads to the creation of reactive oxygen species.^[^ ^4^ ^]^ The resistance of nitroarenes to the oxidative degradation (the common degradation pathway) is increased by the high stability of the aromatic ring, and the total effect is the bioaccumulation of these compounds in the environment, especially in water and soil.^[^ ^1^ ^]^ For these reasons, the discharge of these compounds should be avoided, whereas a revaluation of these wastes is highly desirable.

Catalytic hydrogenation to substituted aniline represents the most common method for the valorization of waste nitroarenes.^[^ ^1^, ^2^, ^5^, ^6^, ^7^, ^8^, ^9^, ^10^, ^11^ ^]^ This process is of particular interest due to the utility of substituted anilines as starting molecules for the pharmaceutical and dye industries. The active metals employed in nitroarenes hydrogenations are Au,^[^ ^12^ ^]^ Pd,^[^ ^13^ ^]^ and Pt.^[^ ^14^ ^]^ However, the high cost of these elements has prompted research into the use of less noble metals such as Fe, Ni, and Cu.^[^ ^3^ ^]^ Iron, in particular, is one of the most abundant elements on Earth, is highly biocompatible and has a long history of use as a reducing agent for nitrogen compounds (Béchamp reaction and Haber–Bosch process are just two examples). Typical reducing agents employed in the hydrogenation of nitroarenes are: molecular hydrogen,^[^ ^15^ ^]^ formic acid (which can also be readily obtained from biomass or CO_2_),^[^ ^16^ ^]^ sodium borohydride,^[^ ^17^, ^18^, ^19^, ^20^ ^]^ and hydrazine.^[^ ^3^ ^]^ The combined use of hydrate hydrazine and iron‐based catalysts has received great attention for the hydrogenation of nitroarenes,^[^ ^21^, ^22^, ^23^, ^24^, ^25^ ^]^ as it results in the generation of only N_2_ and H_2_O as by‐products. In regard to iron‐based catalysts, they are typically composed of an iron species supported on an inorganic or organic matrix.^[^ ^26^, ^27^, ^28^ ^]^

Steel slags (SS) are by‐products of steel factories composed mostly of hydroxides, oxides, carbonates, and silicates of metals, such as sodium, calcium, aluminum, magnesium, and iron, among others. The disposal of SS in soils or caves is of high concern due to their alkalinity which can promote the unnatural mobility of elements that would otherwise be immobilized under neutral environmental conditions. Recently, the employment of SS in catalysis has been developed for organic reactions occurring under basic pH values.^[^ ^29^ ^]^

In this study, the catalytic activity of several iron‐supporting SS, prepared starting from iron(II) and iron(III) sources for the conversion of nitroarenes into the corresponding anilines, was evaluated. The screening demonstrated that the catalyst prepared using FeCl_3_·6H_2_O was composed of 2L‐ferrihydrite supported onto SS and was outstanding in terms of efficiency and recyclability. The novelty of this work lies in the experimental evidence of a new high‐catalytically active material obtained by simply mixing an aqueous iron salt with highly basic SS, thus avoiding the addition of any external base^[^ ^3^ ^]^ and/or further treatment (calcination, hydrothermal process, etc.). This approach makes the entire system highly appealing from both sustainability and circular economy perspectives.

## 2 Experimental Section

### 2.1 Materials and Methods

All chemicals were purchased from commercial suppliers and used as received. The SS investigated in this study (referred as **SS**) were produced between 1972 and 1980 by the steel factory “ILVA” (Taranto, Italy) and subsequently disposed of in an open‐air landfill for a period exceeding 40 years. The **SS** were sieved (80 mesh) before use. Elemental analyses were carried out using a portable energy dispersive X‐ray fluorescence spectrometer (P‐XRF, NITON XL3t, Thermo Scientific, Waltham, USA) equipped with an Ag collimator source (50 KeV and 40 µA), and a large SSD detector (energy resolution <160 eV @Mn–Kα). X‐ray powder diffraction data were collected in air using a PANalytical Empyrean X‐ray diffractometer with Bragg–Brentano geometry, large beta filter‐Nickel, detector (PIXcel3D), and CuKα radiation, operating at 40 kV/40 mA. The powder X‐ray data were collected in the 2θ range of 10° to 90° (step size of 0.026, scan step time of 996.54 s). The diffraction patterns were processed using the PANalytical B.V. software HighScore Plus version 3.0e. Scanning electron microscopy (SEM) analyses were carried out by employing an FESEM Zeiss sigma 300 VP (Zeiss Oberkochen, Germany) operating at 15 kV. The samples were obtained spreading little amount of sample powder on a conductive carbon pad on the specimen holder. Scanning transmission electron microscopy (STEM) analyses were carried out using a field emission scanning electron microscope (Nova NanoSEM 450, FEI Company) equipped with an STEM II detector and operating at 30 kV. The sample preparation for the STEM analyses was as follows: the sample powder was suspended in distilled water and sonicated for 20 min at room temperature. Subsequently, the samples were prepared by dipping a 200‐mesh carbon‐coated gold microscope grid into the ultrasonicated suspensions and dried. The chemical composition and elemental mapping were determined by energy dispersive X‐ray analysis (EDX) under SEM observation. FT‐IR spectra (in attenuated total reflectance, ATR mode) were recorded on a Jasco FT‐IR 4200 spectrophotometer. ^1^H and ^13^C{^1^H} NMR spectra were recorded on an Agilent 500 spectrometer (125 MHz for ^13^C). X‐ray photoelectron spectroscopy (XPS) analyses were performed with a Versa Probe II Scanning XPS Microprobe spectrometer (Physical Electronics GmbH) using a monochromatized AlKα source with an X‐ray spot size of 200 mm and a power of 47.6 W. Wide scans and detailed spectra were acquired in fixed analyzer transmission mode with a pass energy of 117.40 and 46.95 eV, respectively. An electron gun was used for charge compensation (1.0 V, 20.0 mA). All binding energies were referenced to C1s at 284.8 ± 0.1 eV for adventitious hydrocarbon. Data processing was performed using MultiPak software v. 9.9.0.8, 2018. Each analysis was performed in three different sample points. UV–vis spectra were recorded on a dual beam Jasco V‐670 spectrophotometer. Absorbance was measured in the 280 ÷ 750 nm range, using a quartz cuvette (optical path 10 mm). GC‐MS data (EI, 70 eV) were acquired on an HP 6890 instrument using an HP‐5MS cross‐linked 5% phenylmethylsiloxane (30.0 m × 0.25 mm × 0.25 mm) capillary column coupled with a mass spectrometer HP 5973. FT‐IR spectra (in attenuated total reflectance, ATR mode) were recorded on a Jasco FT‐IR 4200 spectrophotometer. GLC analyses were performed using an HP 6890 instrument equipped with FID detector and an HP‐1 (Crosslinked Methyl Siloxane) capillary column (60.0 m × 0.25 mm × 1.0 mm). Conversions and yields were calculated by GLC analysis using biphenyl as the internal standard. All products were identified by comparison of their GLC retention times and MS spectrograms with those of authentic samples (Sigma‐Aldrich). Room Temperature Mössbauer experiments were performed using a constant acceleration spectrometer operating in transmission geometry, mounting a ^57^Co source in Rh matrix, nominal strength 1850 MBq. The sample was prepared by mixing 100 mg of crushed powder in petroleum jelly. The spectra were fitted to Lorentzian line shape with the statistical best fit evaluated by the reduced *χ* ^2^ method. The hyperfine parameters, isomer shift (*δ*), quadrupole splitting (Δ) or quadrupole shift (*ε*), when magnetic coupling is present, half linewidth at half maximum (Γ_+_), are expressed in mm s^−1^, while the internal magnetic field (*B*) in Tesla and the relative areas (*A*) in %. The velocity was calibrated against a α‐Fe foil and *δ* is quoted relative to metallic iron at room temperature. Physisorption analysis was performed using Quantachrome Autosorb iQ apparatus. All analyzed samples were previously oven‐dried at 80 ± 0.5 °C for 24 h and then placed in a dryer. The weight of empty and full cell was measured three times before and after outgassing. The outgassing procedure was performed at a maximum target temperature of 60 °C for 3 h at a pressure of 5 Torr. Then, the adsorption/desorption measurements were carried out using N_2_ gas adsorbate with 2 min equilibration times at each point and a bath temperature of −196 °C. Surface area was determined by the multipoint Brunauer–Emmett–Teller (BET) method.

### 2.2 Characterization of Steel Slags

The characterization of the **SS** used as support for the iron has been reported in a previous work.^[^ ^30^ ^]^ Briefly, XRF analysis showed that Ca is the most abundant element (31.2%_w_), followed by Fe (18.8%_w_), Mn (4.7%_w_), and Si (3.8%_w_). EDX mapping exhibited a homogeneous elemental distribution of Ca, Al, and Na together with O. On the contrary, Fe, Mg, and Mn showed an uneven distribution, suggesting a potential correlation between these elements. FT‐IR analysis showed the vibrations for hydroxides, carbonates, and silicates. XRD analysis of **SS** revealed the presence of portlandite (Ca(OH)_2_), calcite (CaCO_3_), quartz (SiO_2_), brownmillerite (Ca_2_(Al,Fe)_2_O_5_), dolomite (CaMg(CO_3_)_2_), larnite (Ca_2_SiO_4_), hematite (a‐Fe_2_O_3_), and wüstite (FeO), along with a significant proportion of amorphous compounds.^[^ ^30^ ^]^

### 2.3 Synthesis of the Catalysts

**Scheme 1:**
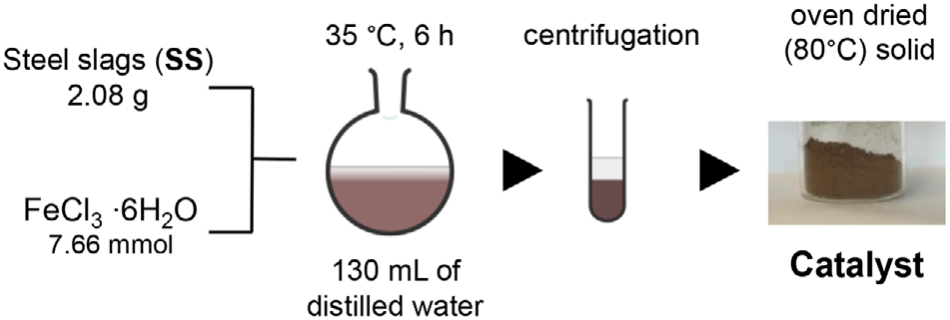
Preparation of **Fe3** catalyst.

Five different iron‐supporting steel slags were prepared, employing FeCl_3_·6H_2_O, FeSO_4_·7H_2_O or FeCl_2_·4H_2_O as iron(III) or iron(II) precursors. A defined quantity of the iron salt(s) was dissolved in 130 mL of deionized water and 2.08 g of sieved steel slags was added to the resulting solution. The mixture was kept under stirring at 35 °C for 6 h, causing a progressive fading of the color. Subsequently, the resulting suspension was centrifuged at 4000 rpm for 15 min and the recovered solid was washed three times with distilled water and oven‐dried at 80 °C overnight. The amount and the type of iron salts employed for the synthesis of the catalysts are detailed in **Table** **1**, with the synthesis of the catalyst prepared from FeCl_3_·6H_2_O being depicted in **Scheme** **1**.

**Table 1:** Amount of iron salts used for the synthesis of the iron‐supporting steel slags. In all cases the specified salts were mixed in water with 2.08 g of sieved steel slags. The weight percentage of iron in the materials was measured by XRF analysis.

Entry	Catalyst	Iron salt			Fe %_wt_
		FeSO_4_·7H_2_O	FeCl_3_·6H_2_O	FeCl_2_·4H_2_O	
1	**FeA** ^a)^	6.00 mmol	3.00 mmol	–	38.1 ± 0.3
2	**Fe2**	7.66 mmol	–	–	25.8 ± 0.9
3	**Fe3**	–	7.66 mmol	–	39.2 ± 0.7
4	**Fe2_Cl**	–	–	7.66 mmol	37.4 ± 1.4
5	**Fe3_low**	–	2.45 mmol	–	25.9 ± 0.1

^a)^ From ref. [2].

### 2.4 Catalytic Runs

In a 25 mL two‐necked round‐bottom flask, the nitroarene (0.50 mmol), the catalyst (40.0 mg), hydrazine monohydrate (5.0 mmol), and the internal standard (0.325 mmol) in 5.0 mL of ethanol were stirred under reflux. The internal standard was biphenyl except for the reaction of 4‐iodonitrobenzene for which *n*‐decane was used. The reactions were monitored taking 0.050 mL at fixed time intervals with a syringe through a silicone septum. Subsequently, each sample was centrifuged at 15 000 rpm for 2 min and then the supernatant phase was analyzed via GLC‐FID. The course of the reactions of 4‐nitrophenol (carried out without internal standard) was monitored by UV–vis. 50 mL of the supernatant phase was dissolved in 1.5 mL of ethanol and 15 mL of this solution was transferred into a quartz cuvette containing 3.0 mL of ethanol and one drop of sodium hydroxide 1.0 m. The absorbances at 314.0 and 402.5 nm were selected for quantification of 4‐aminophenol and 4‐nitrophenol, respectively.

### 2.5 Hot Filtration Test on **Fe3**

The hydrogenation of nitrobenzene using **Fe3** as catalyst was stopped after 5 min and the solution was subsequently analyzed. The degree of conversion was 23.2%. Then, the solid catalyst was hot filtered off (at 80 °C) using a Gooch filter filled with celite. The filtrate was then subjected to stirring at reflux, and the reaction progress was monitored by GLC‐FID. After 0, 30, and 45 min the conversion into aniline was 23.1%, 23.1%, and 23.3%, respectively.

### 2.6 Recycling of the Catalyst

The feasibility of recycling of **Fe3** for the reduction of nitrobenzene was investigated. After the first cycle the reaction mixture was transferred into a vial using acetone to wash the flask walls. Then, the mixture was centrifuged at 4000 rpm for 10 min and the recovered solid was washed with acetone (2 × 5 mL), oven‐dried at 85 °C for 4 h and subsequently employed in a new reaction with fresh reagents. The procedure was repeated for a total of 5 cycles.

### 2.7 Scale‐Up

The **Fe3** catalyzed reduction of nitrobenzene was also tested on a 12.5 mmol scale. Nitrobenzene (1.54 g, 12.5 mmol), **Fe3** (1.00 g), and hydrazine monohydrate (125.0 mmol) in 125 mL of ethanol were stirred in a 250 mL round bottomed flask under refluxing conditions for 1 h. Then, the reaction mixture was filtered using a Gooch filter filled with celite and the ethanol was removed from the filtrate under reduced pressure. Three extractions of the crude with 10 mL of a 1:1 (v/v) solution of ethyl acetate/*n*‐hexane and 10 mL of water permitted to separate aniline from hydrazine. The organic phase was then dried with sodium sulphate, filtrated and the solvent was removed under reduced pressure. Yield: 1.15 g (99%).

## 3 Results

### 3.1 Catalytic Activity of Iron Supporting Steel Slags

Preliminary tests were carried out on the reduction of nitrobenzene to aniline with hydrazine monohydrate (model reaction) without any catalyst (blank reaction, 7% yield, entry 1 of **Table** **2**), with the **SS** as received (8% yield, entry 2 of Table 2) and with the steel slags (**SS**) calcined in air at 400 °C for 3 h (**SS_calc**) (8% yield, entry 3 of Table 2). Therefore, no catalytic activity can be attributed to the iron (or other metals) present in the steel slags, fresh or calcined.

**Table 2:** Hydrogenation of nitrobenzene (0.50 mmol) to aniline with hydrazine monohydrate (5.0 mmol) in ethanol (5.0 mL) at reflux.

Entry	Catalyst	Mass of catalyst [mg]	Time [h]	Yield [%]
1	–		3.0	7
2	**SS**	81	3.0	8
3	**SS_calc**	81	3.0	8
4	**FeA**	40	1.25	>99
5	**Fe3**	40	0.5	>99
6	**Fe2**	40	3.0	>99
7	**Fe2_Cl**	40	1.0	14
8	**Fe3_low**	40	6.0	>99
9^a)^	**Fe3**	1000	1.0	99
10^b)^	**Fe3**	40	1.5	>99

^a)^ 12.5 mmol of nitrobenzene and 125 mmol of hydrazine monohydrate in 125 mL ethanol were used;

^b)^ 0.50 mmol of nitrobenzene and 1.0 mmol of hydrazine monohydrate were used.

With the aim of synthesizing magnetite (Fe_3_O_4_)‐supported **SS**, which could potentially represent a very active and easily recoverable (by magnetic removal) catalyst for the reduction of nitroarenes,^[^ ^31^, ^32^, ^33^ ^]^ a material (**FeA**) was prepared starting from a solution of FeCl_3_·6H_2_O and FeSO_4_·7H_2_O in a 1:2 molar ratio (entry 1 in Table 1) mixed with **SS**, which caused the easy precipitation of the iron(II)/(III) hydroxides onto it without adding any external base, thanks to its alkaline features.^[^ ^2^ ^]^ The XRD analysis of this material did not show any peak ascribable to magnetite. However, **FeA** was found to be active and recyclable, and quantitative conversion of nitrobenzene to aniline was achieved in the reaction with hydrated hydrazine after 75 min at reflux in ethanol (entry 4 in Table 2). In order to study the effect of the precursor iron species in catalysis, we decided to investigate the catalytic activity of three new materials, prepared by treating steel slags with different Fe(II) and Fe(III) sources (Table 1).

**Figure 1:**
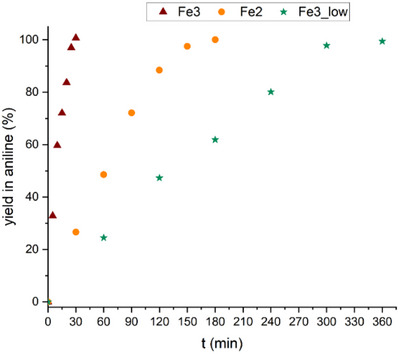
Reaction course for the hydrogenation of nitrobenzene with hydrazine in the presence of **Fe3**, **Fe2**, and **Fe3_low** catalysts. Conditions: nitrobenzene (0.50 mmol); hydrazine monohydrate (5.0 mmol) in ethanol (5.0 mL) at reflux.

The first and the second new materials were synthesized, one starting only from FeSO_4_·7H_2_O (**Fe2**, entry 2 in Table 1) and one starting only from FeCl_3_·6H_2_O (**Fe3**, entry 3 in Table 1), keeping the moles of employed iron constant (7.66 mmol). **Fe3** allowed the quantitative conversion of nitrobenzene to aniline in only 30 min (**Figure** **1** and entry 5 in Table 2), while **Fe2** required 180 min to reach reaction completion (Figure 1 and entry 6 in Table 2). This screening reveals that **Fe3** acted as the most active catalyst, among those investigated. Nevertheless, **Fe2** was also catalytically active, indicating that Fe(II) precursor can also be used to form efficient catalysts. In order to verify whether the lower activity of the catalyst prepared using the Fe(II) salt, in place of Fe(III), was due to the different anions of the iron source (chloride for Fe(III) and sulphate for Fe(II)), the third iron‐supporting steel slag material was prepared using FeCl_2_·4H_2_O as iron source (instead of FeSO_4_·7H_2_O). This material, labeled **Fe2_Cl (**entry 4 in Table 1), was even less active than **Fe2,** giving only 14% yield in aniline after 1 h reaction (entry 7 in Table 2). For comparison, the yield obtained after 1 h of reaction with **Fe2** was 49% (Figure 1). Further tests were carried out with a material prepared by loading the **SS** with a lower amount of FeCl_3_·6H_2_O (**Fe3_low**, entry 5 in Table 1). In this case 6 h were required to achieve a quantitative yield of aniline (Figure 1 and entry 8 in Table 2).

To test the scalability of the model reaction, the hydrogenation of nitrobenzene was carried out with 25 times larger amounts of reagents. Thus, employing **Fe3** as a catalyst a 99% isolated yield of aniline was recorded after 1 h reaction (entry 9 in Table 2). This demonstrates the scalability of the process.

A further test was carried out by reducing the amount of hydrazine monohydrate to 1.0 mmol (entry 10 in Table 2) and, using **Fe3** as catalyst, a quantitative conversion of nitrobenzene to aniline was observed after 1.5 h. Thus, even when the amount of reducing agent used was reduced to a slightly superstoichiometric level, the process was still efficient.

### 3.2 Scope of the Reaction

The scope of the reaction was explored using **Fe3** as a catalyst and the results are collected in **Table** **3**. Halonitrobenzenes were easily hydrogenated to the corresponding anilines. Quantitative yields were obtained after 30 min for 4‐chloronitrobenzene (entry 3) and 4‐bromonitrobenzene (entry 4) without hydrodehalogenation. In the cases of 4‐fluoronitrobenzene and 4‐iodonitrobenzene quantitative conversions were registered after 60 min. Interestingly, 4‐iodonitrobenzene gave a quantitative yield in 4‐iodoaniline (without hydrodehalogenation,^[^ ^9^ ^]^ entry 6), while 4‐fluoronitrobenzene (entry 2) gave a small amount of nitrobenzene as a by‐product. Among the nitrotoluenes, only the *ortho*‐substituted compound (entry 8) gave a quantitative yield in the corresponding aniline after 60 min; 4‐nitrotoluene gave 76% yield in 4‐toluidine, while 3‐nitrotoluene was more sluggish, giving 63% yield after 60 min. As expected, the reaction of 2‐nitroanisole was faster than that of 3‐nitroanisole. In fact, 2‐nitroanisole gave quantitative yield after 30 min (entry 13) while 3‐nitroanisole gave 96% yield after 60 min. Finally, 4‐nitrophenol was reduced to 4‐nitroaniline with 69% yield after 60 min (entry 17). 2‐Toluidine is used in forensic chemistry while 4‐aminophenol is a precursor for the synthesis of paracetamol.

**Table 3:** Hydrogenation reactions of nitroarenes to the corresponding anilines.

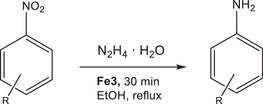			
Entry^a)^	Substrate	Time [min]	Yield [%]
1	4‐Fluoronitrobenzene	30	90
2	4‐Fluoronitrobenzene	60	96
3	4‐Chloronitrobenzene	30	99
4	4‐Bromonitrobenzene	30	99
5	4‐Iodonitrobenzene	30	93
6	4‐Iodonitrobenzene	60	>99
7	2‐Nitrotoluene	30	60
8	2‐Nitrotoluene	60	97
9	3‐Nitrotoluene	30	30
10	3‐Nitrotoluene	60	63
11	4‐Nitrotoluene	30	50
12	4‐Nitrotoluene	60	76
13	2‐Nitroanisole	30	99
14	3‐Nitroanisole	30	82
15	3‐Nitroanisole	60	96
16	4‐Nitrophenol	30	46
17	4‐Nitrophenol	60	69

^a)^ Conditions: 0.50 mmol of nitroarene, 5.0 mmol hydrazine monohydrate, 40 mg of **Fe3** in ethanol (5.0 mL) at reflux.

These results show that nitrobenzenes substituted with electron‐withdrawing groups (halogens in our case) react faster than substrates endowed with electron‐donor groups.^[^ ^34^ ^]^ In the studied *ortho*‐substituted substrates (2‐nitrotoluene and 2‐nitroanisole) the electronic effect prevails on the steric hindrance.

### 3.3 Recycling Test

**Figure 2:**
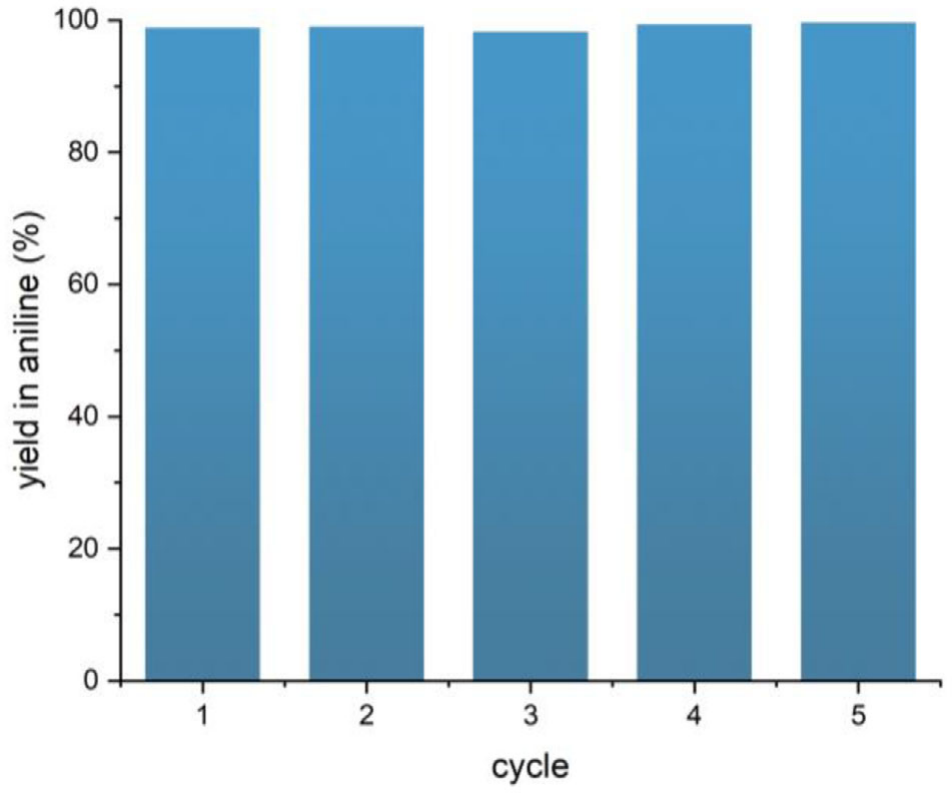
Recyclability of **Fe3** catalyst over five consecutive reaction cycles. Reaction conditions: 0.50 mmol nitrobenzene, 40.0 mg of **Fe3**, 5.0 mmol hydrazine monohydrate, 5.0 mL of ethanol, time = 30 min.

**Figure 3:**
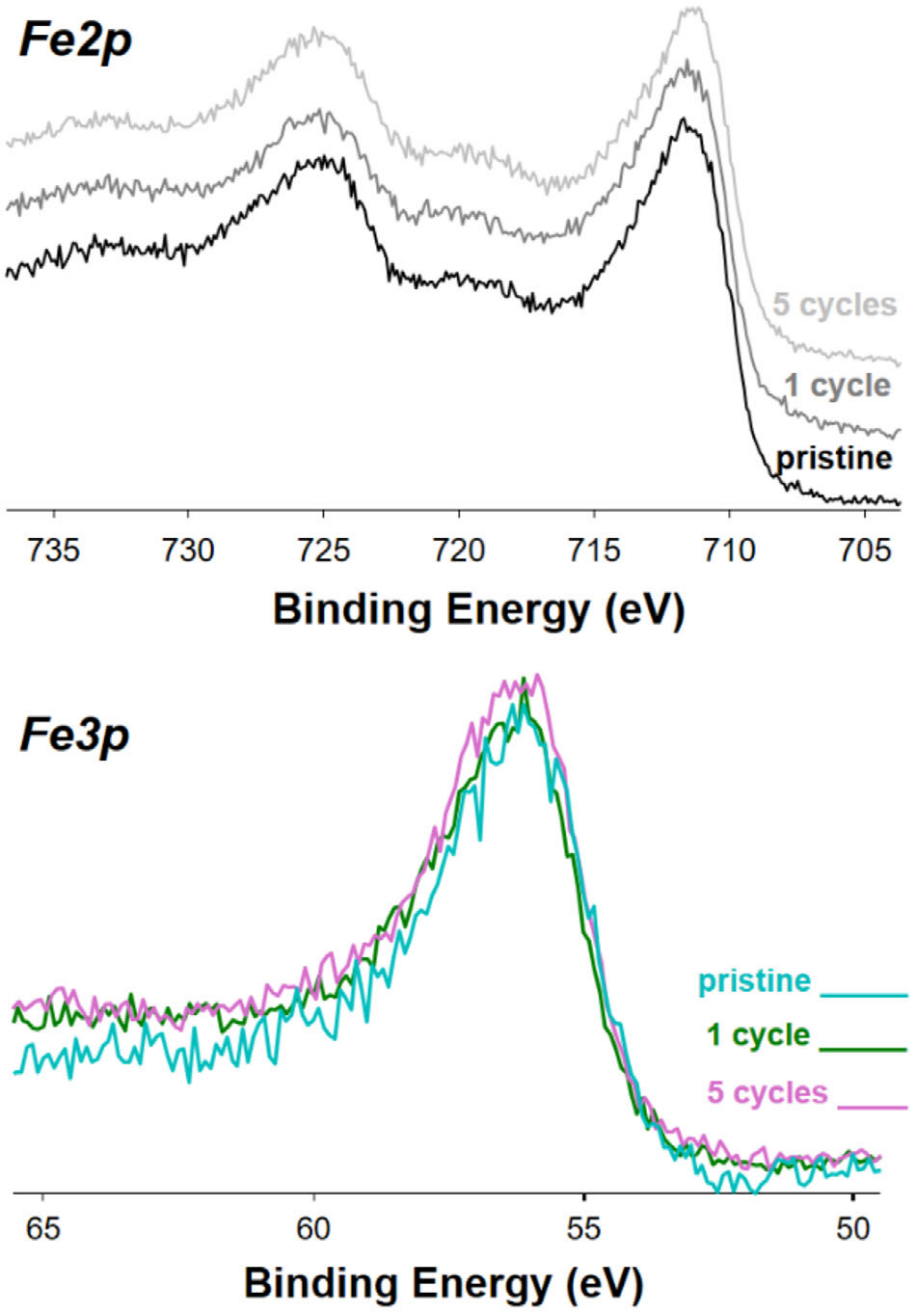
Fe2p (top) and Fe3p (bottom) XP spectra of **Fe3** (pristine, after 1 cycle and after 5 cycles).

The recyclability of the **Fe3** was investigated by reusing the same batch of catalyst for five subsequent consecutive cycles. The results, shown in **Figure** **2**, demonstrate that quantitative yields were achieved for all cycles, with no loss of catalytic activity. XRF analysis indicated that there was no leaching of iron during duty. In fact, the concentration of iron in the catalyst after 1 cycle (40.5 ± 0.3%_w_, Table S2, Supporting Information) was substantially the same as that of pristine **Fe3** (39.2 ± 0.7%_w_, entry 3 of Table 1). The absence of iron leaching in solution during catalysis was also confirmed by XPS. The atomic percentages of the elements detected by XPS on the surface of **Fe3** catalyst before duty, after 1 cycle and after 5 cycles (**Table** **4**) revealed that the amount of iron present on the surface remained constant over the cycles. Fe2p and Fe3p XP spectra of the **Fe3** catalyst before duty, after 1 cycle and after 5 cycles, showed that in all cases iron is present in the oxidation state +3 and in a form ((oxy)hydroxide species, vide infra) which is the same before and after catalysis (resting state). In fact, the line shape and the binding energy (BE) of the Fe2p and of Fe3p signals (**Figure** **3**) are identical for all the samples considered, as well as FT‐IR (in ATR mode) of the **Fe3** catalyst recorded before and after duty (Figure S33, Supporting Information).

**Table 4:** Atomic elemental percentages detected by XPS analyses on pristine and recycled **Fe3** catalyst.

Fe3	%Fe	%C	%O	%Si	%Ca
Pristine	18.7 ± 1.2	8 ± 1	63 ± 1	8.6 ± 0.5	2.6 ± 0.5
After 1 cycle	15.7 ± 1.3	19 ± 7	54 ± 4	7.9 ± 0.8	2.3 ± 0.5
After 5 cycles	16.0 ± 2.0	19 ± 6	55 ± 4	7.1 ± 0.5	2.2 ± 0.5

### 3.4 Heterogeneity of the Catalysis

In order to study the heterogeneity of the catalysis, a hot filtration test was carried out on the model reaction catalyzed by **Fe3**, as described in the Experimental Section.^[^ ^35^, ^36^ ^]^ After hot filtration, and in the absence of **Fe3**, the conversion stopped at the value registered before filtration, indicating that the hot filtrated solution was catalytically inactive. This result, together with the absence of iron leaching in solution discussed above, indicates that the catalysis is truly heterogeneous.

### 3.5 Characterization of the Catalysts

#### 3.5.1 SEM/EDX and XRF Analysis

**Figure 4:**
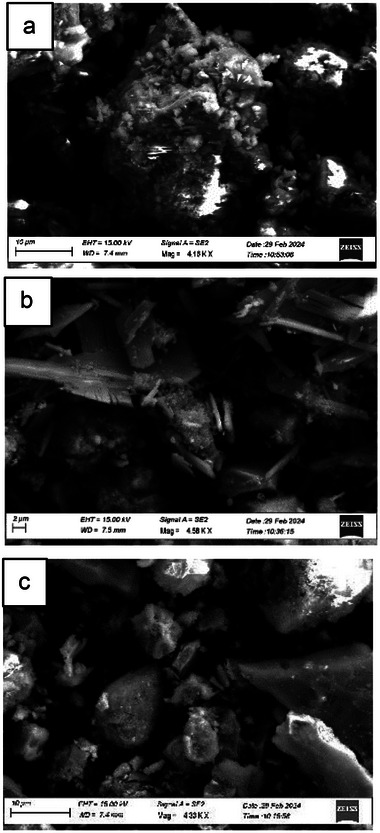
SEM images of a) **SS**, b) **Fe2**, and c) **Fe3**.

The SEM images of the powders of the different samples, **Fe2** and **Fe3** along with the unloaded **SS**, are shown in **Figure** **4**. The image of **SS** (Figure 4a) showed a polydisperse material composed of aggregated particles and grains of different dimensions, as commonly found in metallurgical slags. At a morphological level, **Fe2** (Figure 4b) revealed the copresence of two components: small and aggregated particles, and long and narrow aggregated rods with a defined crystalline habitus. **Fe3** particles (Figure 4c) are constituted by well separated grains.

The elemental composition of the prepared materials as determined by XRF analysis is given in **Table** **5**. It is evident that the Fe weight percentage increases in the order **SS** < **Fe2** < **Fe2_Cl** ≈ **Fe3** (18.8 < 25.8 < 37.4 ≈ 39.2), while the Ca weight percentage decreases in the order **SS** > **Fe2** > **Fe2_Cl** > **Fe3** (31.2 > 16.6 > 11.1 > 9.0). It is interesting to note that S percentage was negligible in all the catalysts (as well as in the pristine **SS**), except for **Fe2** where S = 14.8_%_ _wt_. The remaining elements (Si, Mn, and Cr) remained almost unchanged, with a slight decrease registered only for **Fe2**.

**Table 5:** Elemental analysis (wt%) of the investigated iron‐supporting steel slags carried out by XRF analysis.

Element	**SS**		**Fe2**		**Fe3**		**Fe2_Cl**	
	wt%	SD	wt%	SD	wt%	SD	wt%	SD
**Fe**	18.8^a)^	1.4	25.8	0.9	39.2	0.7	37.4	1.4
**Ca**	31.2^a)^	1.8	16.6	0.4	9.0	0.10	11.1	0.3
**S**	0.17	0.03	14.8	0.17	<LOQ		<LOQ	
**Si**	3.8^a)^	0.2	2.5	0.2	3.8	0.2	2.7	0.1
**Cr**	0.11	0.01	0.08	0.01	0.10	0.01	0.09	0.01
**Mn**	4.7^a)^	0.4	3.5	0.3	4.4	0.3	4.2	1.2
**Balance**	40.4	2.3	36.7	1.6	42.8	1.1	44.5	1.94

^a)^ From ref. [30].

**Figure 5:**
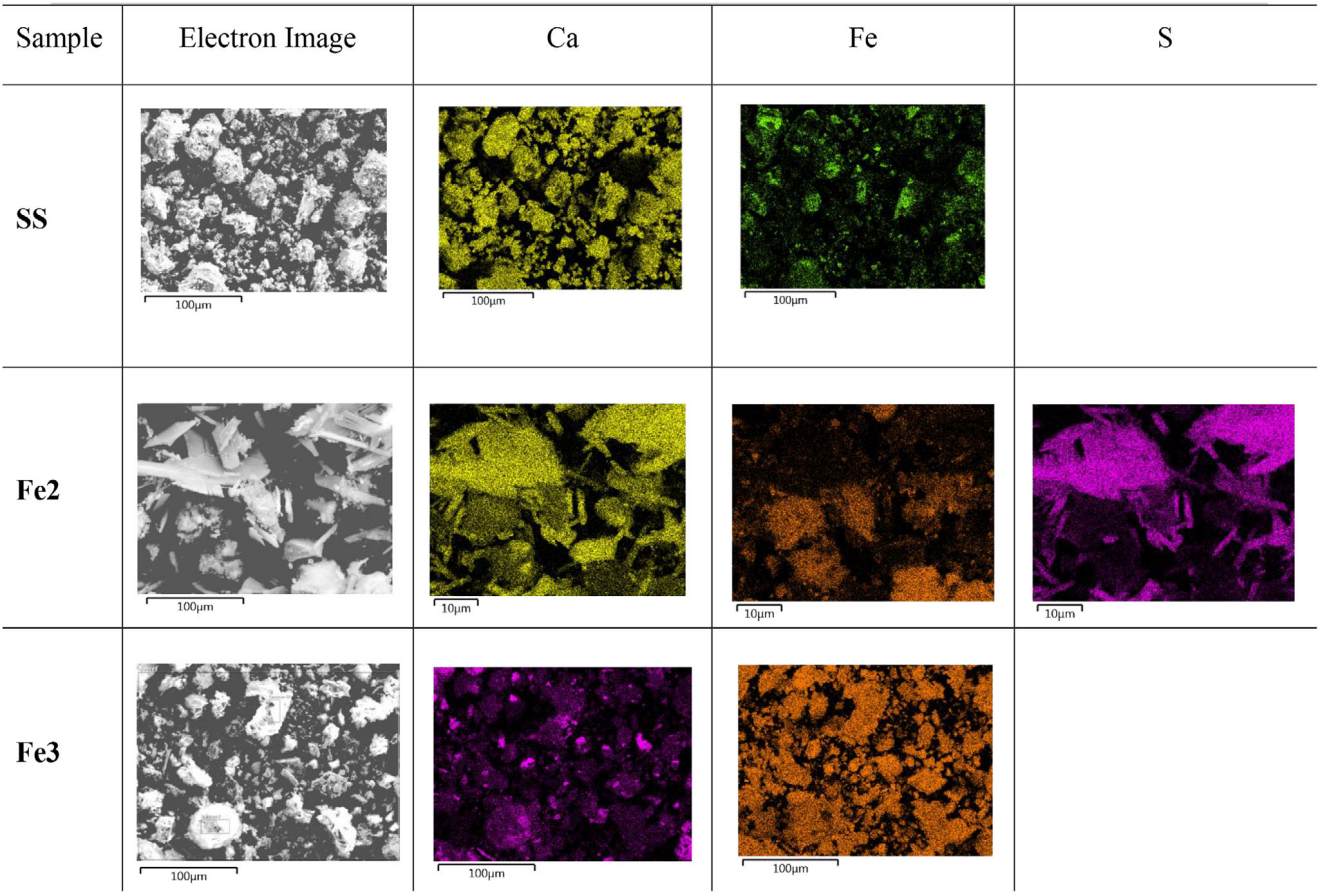
Elemental dispersion of calcium, iron and sulfur on iron‐supporting steel slags as revealed by EDX mapping.

EDX mapping of the prepared materials (**Figure** **5**) shows that iron is unevenly dispersed in **SS** and in **Fe2**. In the latter material, iron appears to be absent in the long and narrow aggregated rods, whereas **Fe3** shows a homogeneous distribution of iron. Moreover, calcium, which is homogeneously dispersed on **SS** became more concentrated onto the rod‐shaped particles in **Fe2** and unevenly dispersed on **Fe3**. Sulphur, which was not detected in **SS** and in **Fe3**, was present in large amounts in **Fe2** (consistently with by XRF analysis). Interestingly, the sulfur distribution in **Fe2** is superimposable with the areas of highest calcium concentration, corresponding to the rod‐shaped crystalline particles. In addition, Fe is absent in such S‐bearing crystals (Figure 5).

#### 3.5.2 XRD Analysis

**Figure 6:**
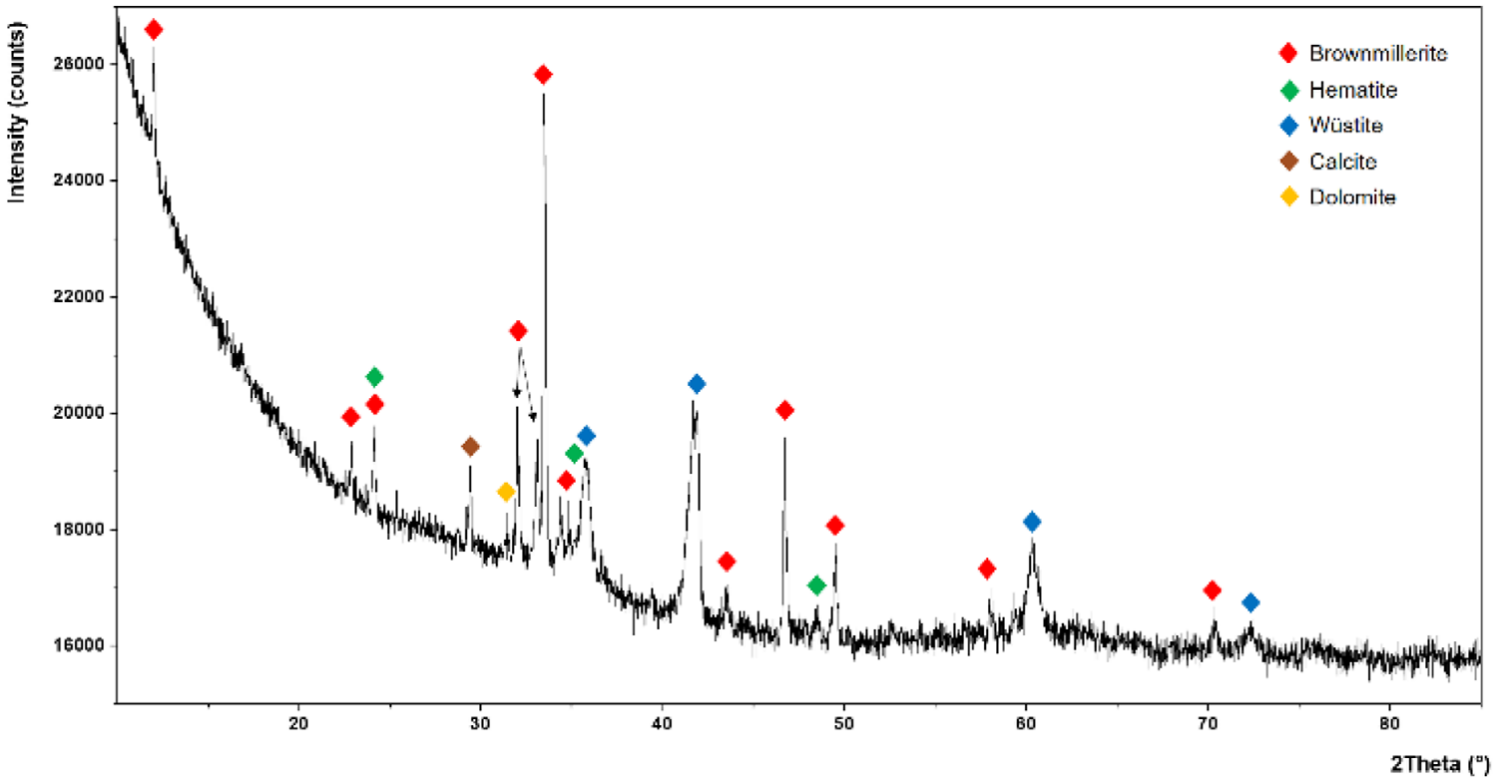
XRD pattern of **Fe3**.

**Figure 7:**
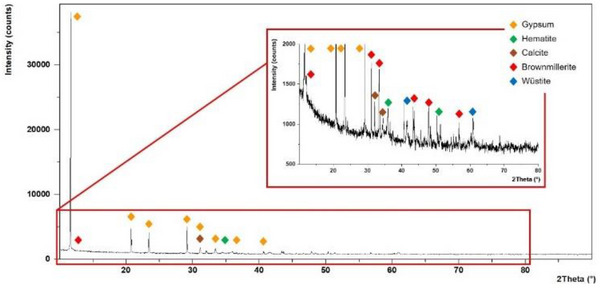
XRD pattern of **Fe2**.

XRD patterns of the iron‐supporting steel slags **Fe3** (**Figure** **6**), **Fe2** (**Figure** **7**), and **Fe2_Cl** (Figure S34, Supporting Information) were recorded. In all cases, no new iron‐containing crystalline phase was visible in the catalysts with respect to pristine **SS**. This suggests that the iron species formed on the steel slags catalyzing the hydrogenation of nitroarenes are noncrystalline (amorphous). In the case of **Fe2**, the diffractogram is dominated by the peaks of gypsum (CaSO_4_·2H_2_O, Figure 7), suggesting that the treatment of the alkaline steel slags with FeSO_4_·7H_2_O triggers its reaction with calcium hydroxide, producing insoluble amorphous iron(II) hydroxides (enlarged insert in Figure 7) and crystalline calcium sulphate, which are deposited on the steel slag. Conversely, in the case of FeCl_3_·6H_2_O, its reaction with Ca(OH)_2_ present in the **SS** results in soluble CaCl_2_ and insoluble amorphous iron(III) hydroxides.

This explains why the percentage of Fe in **Fe2** (25.8%, Table 5) is significantly lower than that in the other catalysts (Table 5). In this respect, it can be expected that the acidic FeCl_3_·6H_2_O reacts with all the alkaline compounds in the **SS**, such as CaCO_3_, MgCO_3_, NaOH, Na_2_CO_3_, etc., forming iron(III) hydroxides (or carbonates) and releasing Na^+^, Ca^2+^, and Mg^2+^ (as chlorides) in solution. This behavior could explain: i) the absence of Cl in the iron‐supporting **SS**; ii) the reduction in the calcium content passing from **SS** (31.2% _wt_, Table 5) to **Fe3** (9.0% _wt_, Table 5); iii) the absence of portlandite (see XRD in Figure 6) in **Fe3**. The less pronounced reduction in calcium content passing from **SS** to **Fe2** (16.6% _wt_, Table 5) is obviously due to the deposition of calcium sulphate mentioned above.

#### 3.5.3 XPS Study

XPS analyses were conducted on the iron‐supporting **SS** to gain insight into the species present on the surface of the obtained materials, which may be responsible for the catalytic activity. The surface elemental composition (for each sample analyzed) was substantially homogeneous, as ascertained by XPS atomic percentages collected in **Table** **6**. It is apparent that the amount of surface iron is very low in the as‐received **SS**, which are rich in Ca, C, O, and Na. A small amount of Si and traces of Cl were also detected. No Mn was observed on the surface of the samples. The low amount of iron on the surface might explain the absence of catalytic activity in the model reaction by the steel slags as received (**SS**) (or calcined, **SS_calc**). Moreover, the other data (Section 2.2), together with the information provided by XPS, support the presence of surface carbonates, oxides, and hydroxides of calcium and sodium.^[^ ^30^ ^]^ In **Fe3** and **Fe2_Cl,** the amount of surface calcium is drastically reduced with respect to **SS,** while in **Fe2** significant amounts of Ca and S are present. For all iron‐bearing steel slags, negligible amounts of Cl and a strong decrease of carbon with respect to **SS** were observed.

**Table 6:** XPS atomic percentages. Data are reported as mean values ± 1S (*n* = 3).

Sample	%C	%O	%Na	%Si	%Cl	%S	%Ca	%Fe
**SS**	28 ± 3	50 ± 2	8 ± 2	2.8 ± 0.6	Traces		10 ± 2	1.0 ± 0.4
**Fe2**	10 ± 2	63 ± 2		6.0 ± 1.3	–	4.5 ± 0.5	6.2 ± 0.5	11 ± 3
**Fe3**	8 ± 1	63 ± 1		8.6 ± 0.5	Traces		2.6 ± 0.5	18.7 ± 1.2
**Fe2_Cl**	16 ± 3	58 ± 2		6.0 ± 0.9	0.9 ± 0.5		3.2 ± 0.5	16.5 ± 1.4

**Figure 8:**
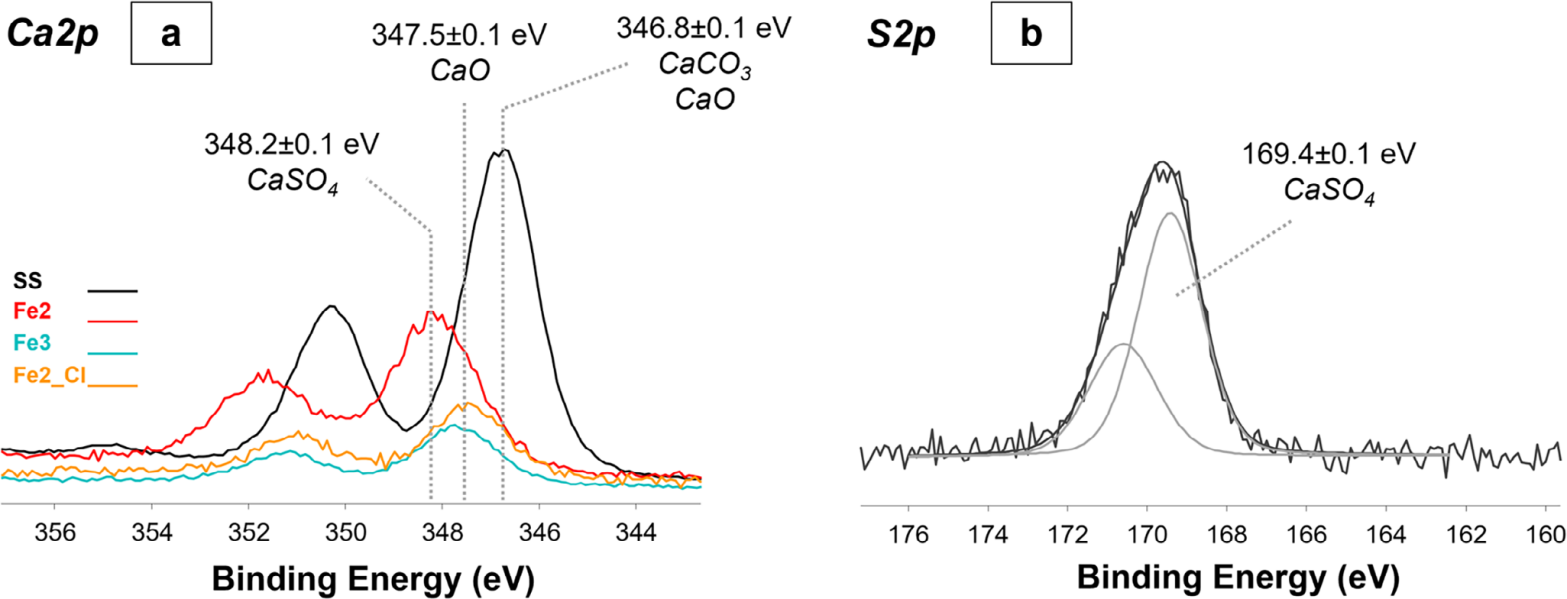
a) Ca2p XP spectra of **SS**, **Fe2**, **Fe3**, and **Fe2_Cl**, and b) curve fitted S2p XP spectrum of **Fe2**.

The Ca2p XP spectrum of **SS** showed only the signals of calcium carbonate (BE = 346.8 ± 0.1 eV) and calcium oxide could not be excluded (BE = 347.5 ± 0.1 eV), while for **Fe3** and **Fe2_Cl** calcium oxide was the only species detected (**Figure** **8**a). In the case of **Fe2**, the characteristic peak of calcium as sulphate (BE = 348.2 ± 0.1 eV, Figure 8a) was recorded, which finds its counterpart in the corresponding S2p XP signal at BE = 169.4 ± 0.1 eV, ascribable to sulphate ion^[^ ^37^ ^]^ (Figure 8b).

Focusing on the differences between **SS** and the prepared materials, the C1s XP spectrum of **SS** (Figure S37, Supporting Information) shows, in addition to organic C coming from unavoidable contamination (BE = 284.8 ± 0.1 eV), an intense signal at BE = 289.4 ± 0.1 eV due to surface carbonates that disappears in the **Fe2**, **Fe3**, and **Fe2_Cl** iron‐supporting steel slags (Figure S37, Supporting Information). Moreover, the Fe2p and Fe3p XP spectra (Figures S38 and S39, Supporting Information) of the studied materials show similar shapes and binding energies for all the iron‐supporting steel slags, indicating the same chemical environment for all the materials.

**Figure 9:**
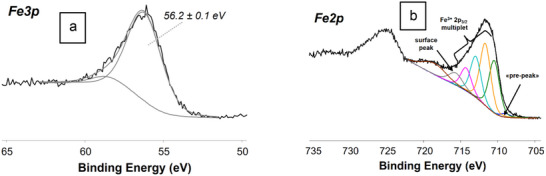
a) Curve fitted Fe3p XP spectrum of **Fe3**, and b) curve fitted Fe2p XP spectrum of **Fe3**.

In particular, the curve fitting of the Fe3p XP spectrum of sample **Fe3** (**Figure** **9**a) resulted in two peak components with the most intense one at BE = 56.2 ± 0.1 eV, indicative of iron(III) species.^[^ ^38^ ^]^ The curve fitting of the Fe2p XP spectrum of **Fe3** (Figure 9b) showed the typical multiplet splitting of Fe^3+^ (BEs = 710.5 ± 0.1 eV, 711.7 ± 0.1 eV, 712.9 ± 0.1 eV, and 714.4 ± 0.1 eV) together with the prepeak and the surface peak at ≈719 eV. In particular, the line shape and the BE values of the multiplet splitting together with the shake‐up‐CG separation (≈8 eV) of this signal indicate that iron is present as Fe(O)OH ((oxy)hydroxy iron(III) species) on the surface.^[^ ^39^ ^]^

The O1s signal of all spectra was curve fitted with three peak components: one at BE = 530.2 ± 0.1 eV, which is characteristic for oxygen of iron(III) oxide; one at BE = 531–532 eV which is characteristic for oxygen of metal hydroxides but also for oxygen of calcium oxide or silicates and sulphates; one at BE = 533.0 ± 0.1 eV which is ascribed to oxygen associated to organic species.

**Figure 10:**
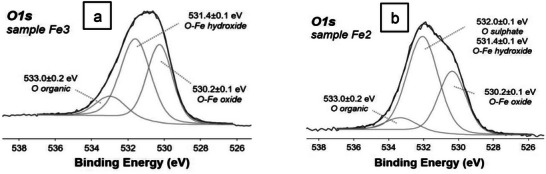
a) Curve fitted O1s XP spectrum of **Fe3**, and b) curve fitted O1s XP spectrum for **Fe2**.

**Figure** **10** shows the O1s curve fitting for **Fe3** (a) and **Fe2** (b). In the O1s XP spectrum of **Fe2** the peak at BE = 532 ± 0.1 eV can be ascribed to both metal hydroxides and sulphate. The atomic ratio between the sum of oxide and hydroxide O and Fe is slightly higher than 2 for all iron‐supporting steel slags (**Table** **7**), thus substantiating the hypothesis that the catalysts contain prevalently (oxy)hydroxide species of iron(III) on the surface.

**Table 7:** XPS atomic percentages of oxygen as oxide (O_oxide_) or hydroxide (O_hydroxide_) and O_oxide+hydroxide_/Fe atomic ratio. Data are reported as mean values ± SD.

Sample	O_oxide_	O_hydroxide_	O_oxide+hydroxide_/Fe
**Fe2**	22 ± 1	33 ± 2	2.3 ± 0.6
**Fe3**	23 ± 1	33 ± 1	2.4 ± 0.2
**Fe2_Cl**	23 ± 5	28 ± 2	2.6 ± 0.3

#### 3.5.4 Mössbauer Spectroscopy

##### Pristine **SS**

**Figure 11:**
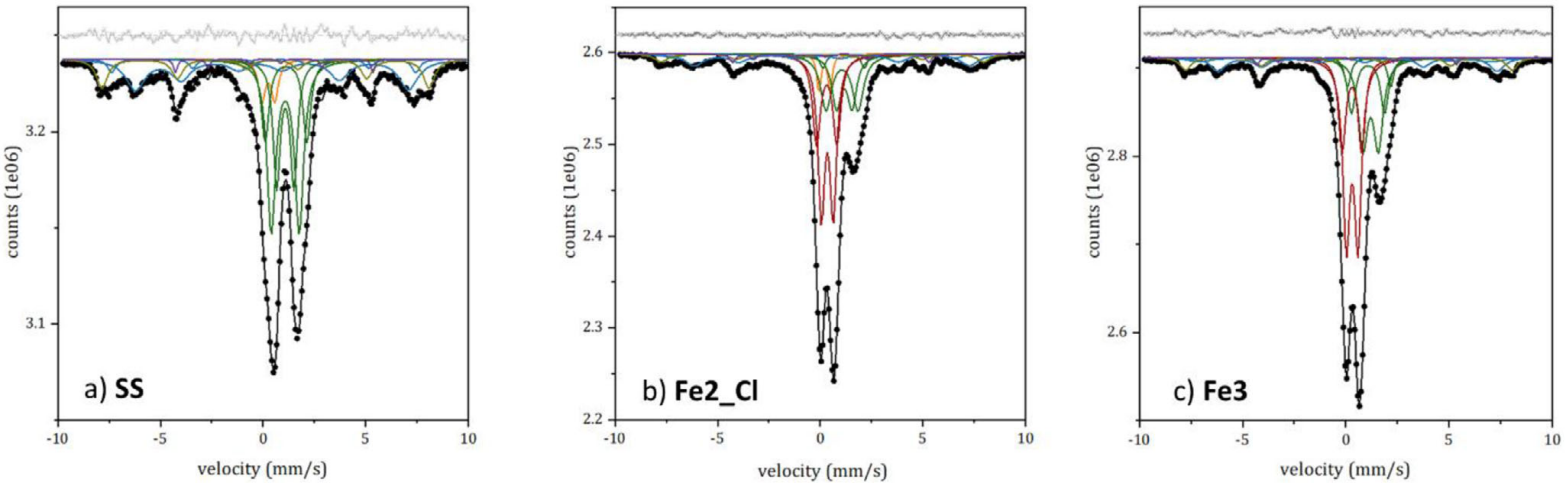
RT Mössbauer spectra of **SS**, **Fe3**, and **Fe2_Cl**. Black dots: experimental data, black line: calculated spectrum. Subspectra: dark green lines for Fe(II) sites, yellow lines for Fe(III)‐4CN, dark red lines for ferrihydrite, dark cyan lines for brownmillerite, dark yellow line for hematite, and violet line for iron carbide.

The Mössbauer spectrum (**Figure** **11**a) collected on **SS** showed an intense and asymmetric two‐line absorption, centered at ≈1.07 mm s^−1^, together with a broad and not well resolved sextet between ≈−7.9 and ≈8.2 mm s^−1^. The spectral features suggest that Fe is distributed in different sites, differing for oxidation state, coordination and magnetic coupling. The best fitting (*χ* ^2^: 1.357) was obtained using an eight‐components model: four doublets and four sextets. The doublets are representative of both Fe(III) and Fe(II) sites: one doublet for Fe(III) and three for Fe(II). The hyperfine parameters of the ferric site, Db 1, are closely related to those of tetracoordinated Fe(III). Despite its low intensity (5% of the total population), it seems to be an effective signal of the spectrum: as a matter of fact, its exclusion drastically increases *χ* ^2^ to 3.664. Concerning the ferrous sites, Fe is distributed on three different sites, differing for Δ values (**Table** **8**). All doublets exhibit half‐linewidths at half maximum (Γ_+_) consistent with crystalline sites, but with a slight broadening. This minor line‐broadening can be attributed to local disorder around ferrous nuclei, rather than to the presence of an amorphous/glassy phase. In the latter case, Fe(II) typically shows Γ_+_ values exceeding 0.4 mm s^−1^.^[^ ^40^ ^]^ A local disorder, arising from different cations among the next nearest neighbors, results in the presence of slightly different ferrous sites, favoring a broadening of the linewidth, suggesting the existence of quadrupole splitting and isomer shift distributions. As mentioned above, the difference in Δ of these ferrous sites reflects a different distortion of the site geometry. Db 2 and Db 3 show a Δ higher than 1.00 mm s^−1^ and can be tentatively attributed to ferrous species hosted in the distorted octahedral sites. Db 2, with a Δ value typical of ferrous ions in silicate network, can be tentatively attributed to Fe(II) hosted in larnite structure.^[^ ^41^ ^]^ Db 3 and Db 4 show parameters close to those observed for Fe(II) in defective wüstite (Fe_1−_ *_x_* O) lattice.^[^ ^41^, ^42^, ^43^ ^]^ The presence of defective wüstite supports the weak doublet ascribed to tetrahedral Fe(III). Acknowledging the presence of wüstite in the pristine steel slag and considering the non‐negligible presence of Mn, the Db 4 doublet could arise from the presence of Mn(II) in the wüstite lattice. As suggested by Gohy et al.,^[^ ^44^ ^]^ the substitution of Fe(II) by Mn(II) results in the existence of two ferrous sites, in addition to the tetrahedral ferric one, with different environments, due to Mn(II), vacancies and Fe(III) ions as nearest neighbors. In this scenario, Db 1, Db 3, and Db 4 could be ascribed to manganowüstite. The magnetic component, i.e., the broad sextet, was interpreted as a superposition of different sextets. According to this model, 39% of the total Fe is distributed over four Zeeman sextets (Sx 1, 2, 3, and 4 in Table 8), which can be tentatively attributed as follows: the sextets with the largest absolute values of *ε* have been attributed to Fe(III) in the tetrahedral and octahedral sites of brownmillerite, Ca_2_Fe_2_O_5_. The data obtained deviate from those related to the pure phase.^[^ ^44^ ^]^ This evidence can be ascribed to the substitution of Fe with other metals, such as Al, Mn, etc.^[^ ^45^ ^]^ It is worth noting that the ratio between the population of tetrahedral and octahedral sites differs substantially from those reported in the literature. This discrepancy can be reasonably ascribed to the superimposition of different signals, due to substitutional effect and/or the presence of other species, which cannot be resolved as consequence of the low intensity of the signals. Moving on to Sx 3, it shows parameters consistent with those of hematite, while Sx 4, with a B close to 29 Tesla, can be attributed to a generic Fe carbide. Due to its low intensity (≈2% of the total Fe population), a more accurate attribution is not possible. The presence of a small percentage of Fe carbide is compatible with the nature of the used material.

**Table 8:** Room temperature hyperfine parameters of **SS**, **Fe2_Cl**, and **Fe3**. Asterisked values were fixed under constraint.

Sample		*δ* [mm s^−1^]	Δ/*ɛ* [mm s^−1^]	Γ_+_ [mm s^−1^]	B [T]	A [%]	Attributions
**SS**	Db 1	0.25 ± 0.07	0.6 ± 0.1	0.19 ± 0.06		5 ± 1	Fe(III)‐4CN in wüstite
	Db 2	1.12 ± 0.02	2.02 ± 0.08	0.23 ± 0.04		13 ± 1	Fe(II)‐6CN in silicate
	Db 3	1.08 ± 0.02	1.4 ± 0.1	0.25 ± 0.03		27 ± 1	Fe(II)‐6CN in wüstite
	Db 4	1.07 ± 0.02	0.83 ± 0.09	0.20 ± 0.03		16 ± 1	Fe(II) in wüstite
	Sx 1	0.14 ± 0.05	0.28 ± 0.04	0.56 ± 0.06	41.2 ± 0.2	21 ± 1	Fe(III)‐4CN in brownmillerite
	Sx 2	0.44*	−0.41 ± 0.03	0.24 ± 0.09	45.9 ± 0.5	4 ± 1	Fe(III)‐6CN in brownmillerite
	Sx 3	0.27 ± 0.04	−0.16 ± 0.04	0.32 ± 0.05	49.4 ± 0.3	12 ± 1	Fe(III)‐6CN in hematite
	Sx 4	0.3 ± 0.1	0.2 ± 0.1	0.11 ± 0.04	29.8 ± 0.5	2 ± 1	Fe carbide
**Fe2_Cl**	Db 4	0.21 ± 0.03	0.53 ± 0.05	0.12*		4 ± 1	Fe(III)‐4CN in wüstite
	Db 1	1.14 ± 0.06	2.02 ± 0.09	0.16 ± 0.06		2 ± 1	Fe(II)‐6CN in silicate
	Db 2	1.05 ± 0.02	1.5 ± 0.1	0.31 ± 0.04		16 ± 1	Fe(II)‐6CN in wüstite
	Db 3	1.15 ± 0.04	0.78 ± 0.1	0.27 ± 0.05		12 ± 1	Fe(II) in wüstite
	Db 5	0.34 ± 0.02	0.57 ± 0.05	0.19 ± 0.01		27 ± 1	Fe(III)‐6CN in ferrihydrite
	Db 6	0.32 ± 0.02	0.96 ± 0.05	0.23 ± 0.01		18 ± 1	Fe(III)‐6CN in ferrihydrite
	Sx 1	0.19 ± 0.05	0.37 ± 0.04	0.50 ± 0.05	41.9 ± 0.3	11 ± 1	Fe(III)‐4CN in brownmillerite
	Sx 2	0.46*	−0.78 ± 0.06	0.3 ± 1	43.3 ± 0.6	3 ± 1	Fe(III)‐6CN in brownmillerite
	Sx 3	0.26 ± 0.06	−0.16*	0.36 ± 0.09	48.7 ± 0.4	5 ± 1	Fe(III)‐6CN in hematite
	Sx 4	0.01 ± 0.04	0.49 ± 0.04	0.16 ± 0.09	26.1 ± 0.1	2 ± 1	Fe carbide
**Fe3**	Db 1	1.13 ± 0.06	2.1 ± 0.1	0.17 ± 0.06		4 ± 1	Fe(II)‐6CN in silicate
	Db 2	1.07 ± 0.03	1.61 ± 0.09	0.22 ± 0.04		10 ± 1	Fe(II)‐6CN in wüstite
	Db 3	1.20 ± 0.04	0.74 ± 0.08	0.27 ± 0.03		19 ± 1	Fe(II) in wüstite
	Db 4	0.31 ± 0.01	0.56 ± 0.06	0.21 ± 0.03		29 ± 1	Fe(III)‐6CN in ferrihydrite
	Db 5	0.29 ± 0.03	0.95 ± 0.07	0.21 ± 0.05		15 ± 1	Fe(III)‐6CN in ferrihydrite
	Sx 1	0.19 ± 0.04	0.36 ± 0.04	0.42 ± 0.07	41.8 ± 0.2	9 ± 1	Fe(III)‐4CN in brownmillerite
	Sx 2	0.44*	−0.48*	0.3 ± 0.1	43.7 ± 0.9	5 ± 1	Fe(III)‐6CN in brownmillerite
	Sx 3	0.31 ± 0.05	−0.17 ± 0.05	0.31 ± 0.07	49.0 ± 0.3	7 ± 1	Fe(III)‐6CN in hematite
	Sx 4	0.01 ± 0.09	0.5 ± 0.1	0.13 ± 0.05	29.9 ± 0.5	2 ± 1	Fe carbide

##### **Fe2_Cl** and **Fe3**

The Mössbauer spectra of **Fe2_Cl** and **Fe3** differ from that of **SS** mainly in the central absorption (Figure 11b,c). These spectra are dominated by an intense and asymmetric doublet centered ≈0.30 mm s^−1^ indicating the presence of paramagnetic/superparamagnetic Fe(III) moieties. Given the absence of other phases containing Fe(III) in paramagnetic regime, as deduced from XRD experiments, the presence of superparamagnetic species is highly probable. The presence of superparamagnetic Fe(III) species suggests the existence of nanosized iron oxide/oxyhydroxide particles. The best fitting was obtained by the model proposed for **SS** with the addition of two doublets (Db 5 and Db 6 in **Fe2_Cl**, Db 4 and Db 5 in **Fe3**). These doublets have hyperfine parameters typical of ferric ions in distorted octahedral geometry. According to literature data^[^ ^46^, ^47^ ^]^ these two doublets can be ascribed to 2L‐ferrihydrite. This hypothesis buttresses the XPS measurements that highlight the presence of Fe(O)OH. According to literature^[^ ^48^ ^]^ these doublets can be ascribed to the ordered particle core (Db 5 in **Fe2_Cl** and Db 4 in **Fe3**) and the other to the more disordered surface layer (Db 6 in **Fe2_Cl** and Db 5 in **Fe3**). The doublet attributed to the surface layer shows higher Δ values compared to those attributed to the core, indicating a higher degree of distortion of the FeO_6_ octahedra at the surface. However, the presence of other iron (oxy)hydroxides cannot be ruled out a priori from the room temperature Mössbauer spectra.

It is noteworthy that the signal of tetrahedral Fe(III) in wüstite (Db 1 in **SS**) is not detectable in sample **Fe3**. Every attempt to fit the spectra with this component gave unsatisfactory results; it is assumable that the overlap between the tiny tetrahedral ferric doublet and the intense one due to ferrihydrite cannot be resolved by a room temperature measurement.

Notably, for both **Fe2_Cl** and **Fe3**, the ratio of Fe(II) to Fe(III) populations in the oxidic phases, excluding the Fe(III) present in ferrihydrite, is similar to that calculated for the pristine **SS** (i.e., 0.7). This observation indicates that the treatment of **SS** with ferric or ferrous chloride does not significantly alter the pristine iron population of **SS**.

#### 3.5.5 STEM Analysis

**Figure 12:**
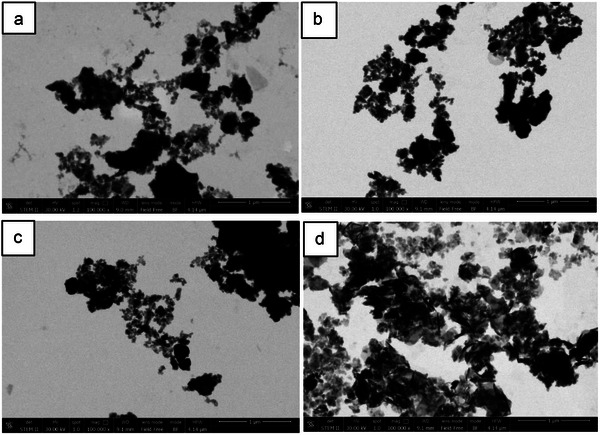
STEM images at magnification 100 000× of a) **SS**, b) **Fe3** pristine, and c) **Fe3** after one catalytic run d) **Fe2_Cl**.

The morphology of the pristine steel slags and of the most representative iron‐supporting **SS** was investigated by STEM analysis. **Figure** **12** shows STEM images of **SS**, **Fe3** (before and after catalysis), and **Fe2_Cl**. Micrometric particles surrounded by sub‐micrometric structures are clearly visible in **SS** and **Fe3** (before and after catalysis) samples. In particular, the scanning transmission electron microscope image (Figure 12a) of the **SS** grain deposited on the gold grid after suspension in water (Figure S40, Supporting Information). The surface of the larger grains is rough and irregular, a morphology common to other types of slag, electric arc furnace slag^[^ ^49^ ^]^ or desulphurization steel slag.^[^ ^50^ ^]^ Although the smaller grains show some small surface features, these appear to be more electron transparent than the hydration products already found in ground granulated blast furnace slag.^[^ ^51^ ^]^ In the specific case of the **SS** studied, it can be hypothesized that the hydration products are due to weathering. The surfaces of such hydration products, which cover the edges of the smaller particles, are very frayed and do not have a well‐defined morphology and are not so repetitive as to suggest the presence of deposited crystalline forms. However, a more defined morphology is evident in **Fe3** (both pristine and after catalysis, Figure 12b,c) compared to that observed in **SS** (Figure 12a).

Iron oxides and hydroxides have a wide variability of morphology and particle size. As reported in many articles, synthetic hematite typically exhibits hexagonal plate, pseudocubic, spindle, and needle morphologies, whereas “kite and tail” and tiny spherical morphologies are typical of goethite and ferrihydrite, respectively.^[^ ^52^, ^53^, ^54^, ^55^ ^]^ In **Fe3** pristine and after one catalytic run (Figure 12b,c), a sub‐micrometric, well‐defined granular morphology is observed as a shell surrounding the larger particles. These granular particles could be attributed to the presence of ferrihydrite particles, similar to those reported in the literature.^[^ ^54^, ^56^, ^57^, ^58^ ^]^ Since this structure is observed for both samples (**Fe3** before and after duty) it can be inferred that the granular morphology remained unchanged after catalysis. In contrast, a sheet‐like morphology typical of a gel‐like structure, is observed for **Fe2_Cl** (Figure 12d), as reported for other aluminosilicate matrices.^[^ ^59^, ^60^ ^]^

#### 3.5.6 Physisorption Analysis

**Figure 13:**
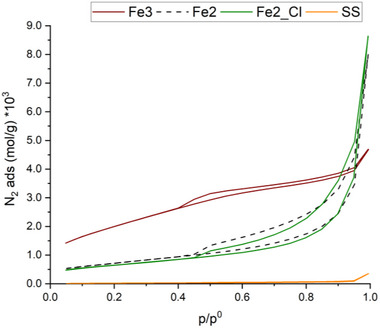
Nitrogen physisorption isotherms for **SS**, **Fe2**, **Fe2_Cl**, and **Fe3**.

Nitrogen sorption analyses were performed on the as‐received **SS**, **Fe2**, **Fe2_Cl**, and **Fe3**. Data recorded for **SS** indicated a very low porosity (≈2 m^2^ g^−1^, **Table** **9**). For the iron‐based catalysts, all the physisorption isotherm obtained (**Figure** **13**) show a hysteresis meaning that capillary condensation occurs inside the pores. The shape of the isotherm obtained when analyzing **Fe3** resembles a composite of Types I and II isotherms, according to the IUPAC classification,^[^ ^61^ ^]^ with an hysteresis loop of type H4. It is classified as type H4 loop not only for the shape of the loop, but also because of the characteristic sharp step‐down in the desorption branch located at p/p^0^ ≈ 0.4. These results indicate a micro–mesoporous material for **Fe3**. Indeed, the more pronounced uptake at low p/p^0^ is associated with the filling of micropores. Regarding **Fe2** and **Fe2_Cl**, the isotherm obtained was similar between the two samples analyzed. In this case, the hysteresis loop is classified as type H3, according to the IUPAC classification.^[^ ^61^ ^]^ The H3 loop is characterized by two distinctive features: an adsorption branch similar to a Type II isotherm (indicating a multilayer adsorption); and a lower limit of the desorption branch located at the expected cavitation‐induced p/p^0^ (p/p^0^ ≈0.4–0.5 for nitrogen at temperatures of 77 K). Loops of this type are given by nonrigid aggregates of plate‐like particles, but also when the pore network consists of macropores, which are not completely filled with pore condensate. The surface areas obtained by the BET method are collected in Table 9. Unfortunately, Barrett–Joiner–Halenda (BJH) cumulative desorption method was not applicable, because in the presence of narrow mesopores, increased surface forces are present and could not be neglected. Similarly, the validity of the Kelvin equation, and therefore of the BJH method, is questionable as the mesopore width decreases because macroscopic considerations can no longer be applied.^[^ ^61^ ^]^ Therefore, the BJH results were not reliable and neither “pore volume” nor “pore radius” were calculated.

**Table 9:** Corrected surface areas of the catalysts.

Sample	BET area [m^2^ g^−1^]	Corrected surface area [m^2^ g^−1^]	Production rate^a)^ [h^−1^]
**SS**	2.18	–	–
**Fe3**	179	517	6.7
**Fe2**	63.6	268	1.1
**Fe2_Cl**	57.3	181	0.5

^a)^ Production rate was calculated as moles of product moles of catalyst^−1^·reaction time^−1^.

The surface areas have been corrected considering the structure of the catalysts. In fact, in our cases, the catalysts are composed of an inert portion (the **SS** support, which is not porous) and an active phase (surface (oxy)hydroxide iron(III) species). Assuming that the deposition of (oxy)hydroxide iron(III) species on the **SS** does not affect the **SS** porosity, we estimated the porosity of the active Fe(O)OH phase, calculated by subtracting the porosity obtained by BET (and related to the whole material) from that of the as‐received **SS** (2.18 m^2^ g^−1^), taking into account the masses involved (see Table S1, Supporting Information). The values of the surface areas thus calculated are referred to as “corrected surface areas” and collected in Table 9. The catalytic activity parallels the corrected surface area of each catalyst, which follows the order: **Fe3** > **Fe2** > **Fe2_Cl**.

## 4 Discussion

Two‐line ferrihydrite^[^ ^62^ ^]^ is a poorly crystalline Fe(III) (oxy)hydroxide that occurs naturally in near‐surface soils and sediments and, due to its mesoporosity, high surface area, and reactivity, has applications in water and air remediation,^[^ ^63^, ^64^, ^65^, ^66^ ^]^ electrochemistry,^[^ ^67^, ^68^ ^]^ and catalysis (mainly nitroarene hydrogenations)^[^ ^34^, ^69^, ^70^, ^71^, ^72^, ^73^, ^74^, ^75^ ^]^ or Fenton‐like oxidations.^[^ ^76^, ^77^, ^78^ ^]^

The synthesis of ferrihydrite is commonly achieved by treatment of aqueous Fe(II)^[^ ^76^ ^]^ or Fe(III) salts with NaOH,^[^ ^79^, ^80^ ^]^ KOH^[^ ^81^ ^]^ or ammonia.^[^ ^82^ ^]^ The present study demonstrates that the treatment of iron(III) or iron(II) aqueous solution with **SS** invariably resulted in the easy synthesis of amorphous (oxy)hydroxide iron(III) species supported on **SS**. The choice of steel slags as a support for the active iron species stems from the known alkalinity of this material,^[^ ^30^ ^]^ which allows the deposition of ferrihydrite by precipitation from aqueous solution, without the addition of an external strong base.^[^ ^28^, ^34^ ^]^

The formation of (oxy)hydroxide iron(III) species follows different pathways, depending on the iron source used: FeCl_3_·6H_2_O reacted with the hydroxides leached out in solution when **SS** are immersed in water^[^ ^30^ ^]^ (or formed by reaction of **SS** basic sites with water) precipitating Fe(III) hydroxides, which dehydrated on the surface of **SS** on oven heating at 80 °C, forming high surface area 2L‐ferrihydrite; FeCl_2_·4H_2_O and FeSO_4_·7H_2_O reacted with the hydroxides forming Fe(II) hydroxides, which are air oxidized to Fe(III) species, which, in turn, form gel‐like structures of (oxy)hydroxide iron(III) species on oven heating at 80 °C; in the case of FeSO_4_·7H_2_O, the formation of Fe(II) hydroxide was accompanied by the formation of CaSO_4_, which also deposited on **SS**.

The different pathways followed for the formation of the (oxy)hydroxide iron(III) species on **SS** have a profound influence on the performance of the obtained catalysts in the hydrogenation of nitrobenzene with hydrated hydrazine. It is known that the activities of iron (oxy)hydroxide species in the hydrogenation of nitroarenes are proportional to their surface areas, suggesting that only the number of available iron sites, and not their structure, determines the catalytic activity.^[^ ^34^, ^72^ ^]^ Therefore, the difference in catalytic activity between **Fe2**, **Fe2_Cl**, and **Fe3**, all of which contain ferrihydrite, can be imputed to a difference in surface area resulting from the specific mechanism followed during their synthesis. This hypothesis was confirmed by BET measurements, which showed that **Fe3** has a micro–mesoporous structure and has the highest surface area (among the catalysts studied). In the case of **Fe2**, the massive presence of crystalline CaSO_4_ with a flat and impermeable surface and with no Fe‐bearing compounds on the surface occludes the pores of the catalyst and results in a lower surface area, which is responsible for the lower catalytic performance compared to **Fe3**. Even worse performance was observed for **Fe2_Cl**, but in this case this may be due to the gel‐like structure of the (oxy)hydroxide iron(III) species revealed by STEM analysis and by the lower surface area obtained by BET.

It is well recognized that the morphology and the consequent catalytic activity of the (oxy)hydroxide iron(III) species formed under aerobic conditions by mixing iron(II) or iron(III) salts with alkaline base solution depends on several parameters, including the oxidation state of the iron source, the anion of the iron salt, the pH, and the temperature.^[^ ^83^, ^84^ ^]^ Indeed, it is known that the use of FeSO_4_·7H_2_O as an iron source leads preferentially to the synthesis of Fe(O)OH in the form of goethite.^[^ ^85^ ^]^ On the other hand, it has been reported that Fe(O)OH in the form of lepidocrocite is the preferred product obtained starting from FeCl_2_·4H_2_O.^[^ ^86^ ^]^ Interestingly, the two crystalline forms of Fe(O)OH showed different catalytic activity in the nitroarene reduction in the presence of hydrazine hydrate, with goethite being more active than lepidocrocite. Therefore, it is predictable that in the complicated systems composed by alkaline **SS** and different iron salts, the kind of the starting metal salt strongly affects the morphology, the surface area and the catalytic activity of the resulting ferrihydrite, with **Fe3** being the material endowed with the highest surface area and, consequently, the best catalytic performance. The absence of Fe on the surface of CaSO_4_ crystals (Figure 5) may indicate that the precipitation of the latter is subsequent to the precipitation of Fe‐bearing compounds on pristine steel slag particles. Such a result highlights the need for a special precipitation route when preparing particle coated catalysts in alkaline media.

The catalyst **Fe3** also showed excellent recyclability: in fact, five subsequent runs could be carried out without loss of activity. No significant modification of the catalyst was detected by XPS, XRF, and FT‐IR. The amount of ferrihydrite onto the surface of the steel slags could be reduced by a factor of three, yielding a material (**Fe3_low**) that was still active in catalysis. Finally, the scalability of the nitrobenzene reduction was proven by carrying out a reaction at the 12.5 mmol scale.

**Scheme 2:**
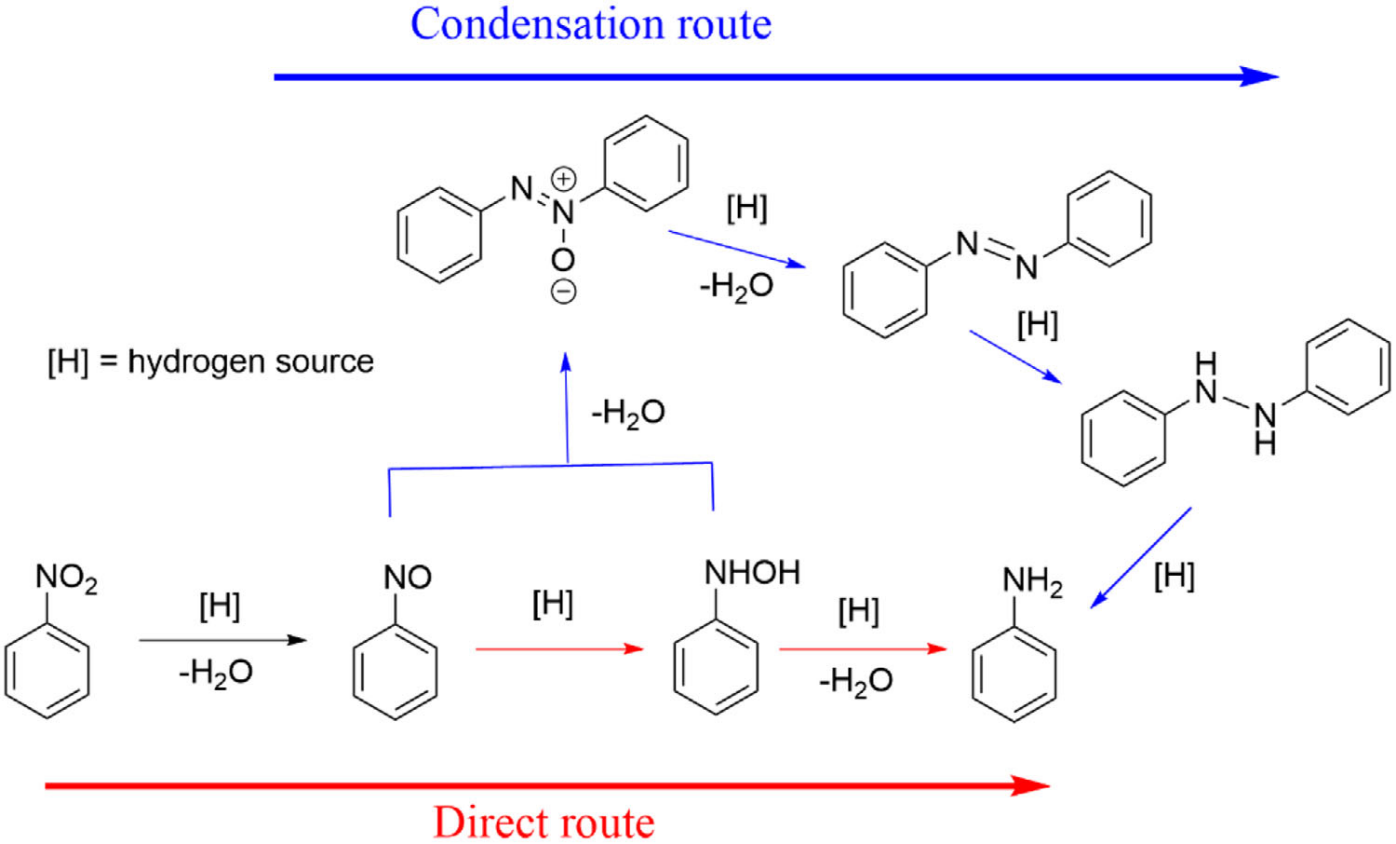
Haber mechanism for nitrobenzene hydrogenation.

**Scheme 3:**
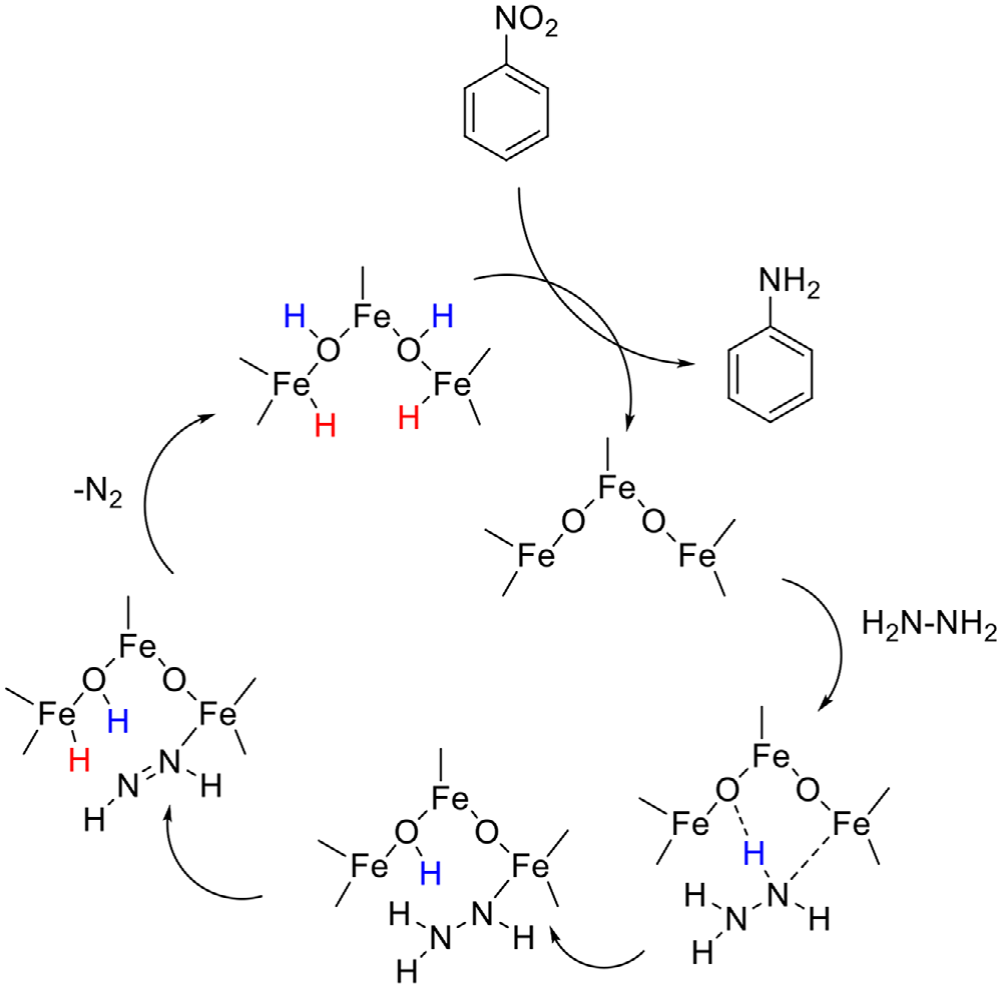
The proposed mechanism of hydrogen active species formation on the surface of **Fe3**.

According to the Haber mechanism, the pathways followed in the hydrogenation of nitrocompounds to anilines are: a direct route, in which nitrosobenzene is formed, followed by the formation of hydroxylaniline and finally aniline; or the condensation route, in which the intermediates of the first route can react together giving life to azoxy compounds that are converted to aniline (**Scheme** **2**).^[^ ^7^ ^]^ Like other reported iron‐based catalytic systems^[^ ^21^, ^25^, ^87^, ^88^ ^]^ using hydrazine hydrate as the reductant,^[^ ^3^ ^]^ the iron‐supported steel slags used in the present work also followed the direct route. In fact, GC‐MS monitoring of the hydrogenation reactions showed that azoxy compounds were never formed in the mixture. Moreover, toxic hydroxylamine was never observed in the catalytic runs, while a very small amount of nitrosobenzene was detected only once during catalytic cycle, ruling out a very recently proposed pathway that skips the formation of nitroso species^[^ ^23^ ^]^ in the direct route and indicates that the rate‐determining step in the **Fe3** system should be the generation of active hydrogen species from the hydrazine reductant^[^ ^89^ ^]^ (**Scheme** **3**). The possible mechanism follows an iron(III)/iron(II) pathway induced by hydrazine and subsequent oxidation of iron(II) to iron(III) with reduction of nitroarene to aniline. In fact, massive formation of magnetite has been observed in the absence of nitroarene. Ferrihydrite has been reported to be the most active iron species among several iron(III) oxides/hydroxides investigated due to its high surface area resulting from its amorphous state.^[^ ^34^, ^72^, ^90^ ^]^ According to literature studies,^[^ ^34^ ^]^ also in the **Fe3** system electron‐withdrawing substituents on the nitroarene ring increased the reaction rate, whereas electron‐donating substituents decreased the reaction rate.

**Table** **10** summarizes the performance of different ferrihydrite catalysts and γ‐Fe_2_O_3_ nanopowder in the benchmark reaction. It is evident that **Fe3** shows very good performance, compared to other literature methods. This is quite pleasing considering that this catalyst is based on an industrial by‐product and is prepared simply by mixing two low‐cost reagents in water, without any careful control of pH or temperature, and without the need for an additional base, as in the other cases reported in Table 10, thus rendering the catalyst preparation procedure appealing from the environmental sustainability point of view.

**Table 10:** Hydrogenation of nitrobenzene to aniline by hydrazine hydrate in ethanol or methanol solvent.

Catalyst	mmol PhNO_2_	mmol Fe	*T* [K]	Time [min]	Production rate^a)^ [h^−1^]	Refs.
**Fe3**	0.50	0.15^b)^	353	30	6.7	This work
MgO/Fe(O)OH	8.1	0.76	333	300	2.1	[71]
Fe(O)OH	10	2.26	338	200	1.3	[72]
Fe(O)OH/hydrazine	1.0	2.25	355	44	0.6	[74]
γ‐Fe_2_O_3_ nanopowder	1.0	0.125	353	80	5.5	[96]

^a)^ Production rate was calculated as moles of product·moles of catalyst^−1^·reaction time^−1^;

^b)^ mmoles of supported Fe(O)OH, not considering the Fe content present originally in **SS**.

Another aspect to be considered in assessing the “greenness” of a catalytic system is the possible production of toxic by‐products. Differently from what happens when using NaBH_4_ as the reductant,^[^ ^91^, ^92^, ^93^, ^94^, ^95^ ^]^ which causes the production of metaborate salt to be disposed, the use of hydrazine monohydrate leads to the formation of only innocuous by‐products, i.e., dinitrogen and water. Since the use of excess N_2_H_4_·H_2_O might be problematic for managing unreacted hydrazine which easily decomposes into ammonia, our efforts were directed also in reducing as much as possible the amount of N_2_H_4_. To our delight, our catalytic system was able to selectively reduce 0.50 mmol of nitrobenzene to aniline in 90 min in the presence of only 1.0 mmol of hydrazine hydrate, thus minimizing the use of reductant (Table 2, entry 10).

## 5 Conclusions

Weathered steel slags coming from industrial sites in Apulia (Italy) are highly alkaline and contain iron species such as wüstite, brownmillerite, and hematite, which have no catalytic activity in the hydrogenation of nitroarenes to anilines. They do, however, have the property of transforming iron(II) or iron(III) salts into amorphous nanocrystalline ferrihydrite, which coats the surface of the steel slag (**SS**) particles and confers to them a remarkable catalytic activity in the abovementioned reaction. Thus, steel slag plays two roles: i) providing the necessary alkalinity to precipitate iron oxides and ii) acting as a robust support for the formed ferrihydrite.

The most active catalyst was **Fe3**, the material obtained using FeCl_3_·6H_2_O as the external iron source, which gave a quantitative yield of aniline (from nitrobenzene) after 30 min of reaction at 78 °C. During catalysis, the catalytically active ferrihydrite did not deactivate and no iron was leached into solution: the catalyst was therefore fully recyclable, retaining its excellent selectivity.

The scope of the hydrogenation was explored using various substituted R–Ph–NO_2_ molecules. The tested halo‐nitrobenzenes (R = F, Cl, Br, I) could be successfully hydrogenated without dehalogenation and reacted faster than substrates endowed with electron–donor groups (R = OH, CH_3_, OCH_3_). Moreover, the *ortho*‐substituted substrates (R = CH_3_, OCH_3_) investigated reacted faster than the *meta*‐substituted ones.

FeSO_4_·7H_2_O and FeCl_2_·4H_2_O also reacted with the hydroxides present in the steel slags to form a shell of ferrihydrite on the **SS** particles. However, the two catalysts obtained (**Fe2** and **Fe2_Cl**) were less active than **Fe3**. The causes of this decrease in activity are ascribed to a poor Fe coverage due to the formation of CaSO_4_ (in the case of **Fe2**) and to the appearance of gel‐like structures on the steel slags surface (in the case of **Fe2_Cl**) that result, in both cases, in a surface area significantly lower than **Fe3**.

This multianalytical investigation has also shown that a complex catalyst such as the one chosen for this study requires in‐depth knowledge of the surface microstructure, which is directly related to catalytic efficiency.

The catalytic system uses a waste material as a catalyst support, does not require any noble metal and retains its efficiency even when using slightly superstoichiometric amounts of hydrazine, thus being consistent with green chemistry principles.

## Conflict of Interest

The authors declare no conflict of interest.

## Supporting information



Supporting Information

## Data Availability

The data that support the findings of this study are available in the Supporting Information of this article.
