# Supplementary material for: Ferrihydrite Supported on Steel Slags as Catalyst for the Hydrogenation of Nitroarenes: A Virtuous Cycle of Wastes
Source: Glob Chall. 2025 Jul 29;9(9):e00201. doi: 10.1002/gch2.202500201 (PMC12418342; doi:10.1002/gch2.202500201)
Supplement: Supplementary file 1 — Supporting Information [file GCH2-9-e00201-s001.docx]

Supporting Information

Ferrihydrite Supported on Steel Slags as Catalyst for the Hydrogenation of Nitroarenes: a Virtuous Cycle of Wastes

*Francesca Derobertis, Maria M. Dell’Anna,* Nicoletta Ditaranto, Luca Nodari, Stefania Liuzzi, Ernesto Mesto, Emanuela Schingaro, Cristina Leonelli, Cecilia Mortalò, Antonino Rizzuti, Carlo Porfido, and Piero Mastrorilli**

F. Derobertis, M. M. Dell’Anna, A. Rizzuti, P. Mastrorilli
Dipartimento di Ingegneria Civile, Ambientale, del Territorio, Edile e di Chimica DICATECh
Politecnico di Bari

via Orabona 4, 70125 Bari, Italy
E-mail: [p.mastrorilli@poliba.it](mailto:p.mastrorilli@poliba.it) [mariamichela.dellanna@poliba.it](mailto:mariamichela.dellanna@poliba.it)

N. Ditaranto
Dipartimento di Chimica and CSGI – Bari Unit
Università degli Studi di Bari Aldo Moro
via Orabona 4, 70125 Bari, Italy

L. Nodari, C. Mortalò
Institute of Condensed Matter Chemistry and Technologies for Energy (ICMATE-CNR)
National Research Council (CNR)
C.so Stati Uniti 4, 35127 Padova, Italy

S. Liuzzi
Dipartimento ARCOD
Politecnico di Bari
via Orabona 4, 70125 Bari, Italy

E. Mesto, E. Schingaro
Dipartimento di Scienze della Terra e Geoambientali
Università degli Studi di Bari Aldo Moro
via Orabona 4, 70125 Bari, Italy

C. Leonelli
Dipartimento di Ingegneria "Enzo Ferrari"
Università degli Studi di Modena e Reggio Emilia
Via P. Vivarelli 10, 41125 Modena, Italy

C. Porfido
Dipartimento delle Scienze del Suolo delle Piante e degli Alimenti Di.S.S.P.A.
Università degli Studi di Bari Aldo Moro
via Orabona 4, 70125 Bari, Italy

Table of contents

[S1. Calibration curves, chromatograms and spectrograms 3](#_Toc192002272)

[S1.1 Aniline 3](#_Toc192002273)

[S1.2 4-Fluoroaniline 5](#_Toc192002274)

[S1.3 4-Chloroaniline 7](#_Toc192002275)

[S1.4 4-Bromoaniline 8](#_Toc192002276)

[S1.5 4-Iodoaniline 10](#_Toc192002277)

[S1.6 2-, 3-, 4-Toluidines 11](#_Toc192002278)

[S1.7 2-, 3-Anisidines 15](#_Toc192002279)

[S1.8 4-Aminophenol 17](#_Toc192002280)

[S2. Calculation of the corrected surface area 19](#_Toc192002281)

[S3. Supplementary characterisation results 20](#_Toc192002282)

[S3.1 FTIR spectra of catalyst Fe3 before and after duty 20](#_Toc192002283)

[S3.2 X-Ray diffraction pattern of Fe2_Cl 20](#_Toc192002284)

[S3.3 XP spectra 21](#_Toc192002285)

[S3.4 Images of catalysts suspensions obtained after sonication 23](#_Toc192002286)

[S3.5 Nitrogen physisorption isotherms of Fe3 before and after one catalytic run 23](#_Toc192002287)

S1. Calibration curves, chromatograms and spectrograms

S1.1 Aniline

Five different solutions were prepared using commercial aniline and biphenyl in 5 mL of ethanol. The method employed at GLC consisted in an isothermal at 70 ºC for 30 sec, followed by an increase of 8 ºC/min until 280 ºC and an isothermal at this temperature for 5 min. The obtained calibration curve is displayed in Figure S1 where the peak area ratio is plotted against the mass ratio. Indeed, A_an_/A_st_ stands for the ratio of the area of the analyte (in this case aniline) peak, and the area of the internal standard (in this case biphenyl) peak; while m_an_/m_st_ stands for the ratio between the weighted mass of the analyte (in this case aniline) and the weighted mass of the internal standard (in this case biphenyl).

**Figure S1.** Calibration curve for the quantification of aniline.

**Figure S2.** GLC plot of the sample retrieved from the reaction mixture after 30 min of reaction in which **Fe3** was used as catalyst.


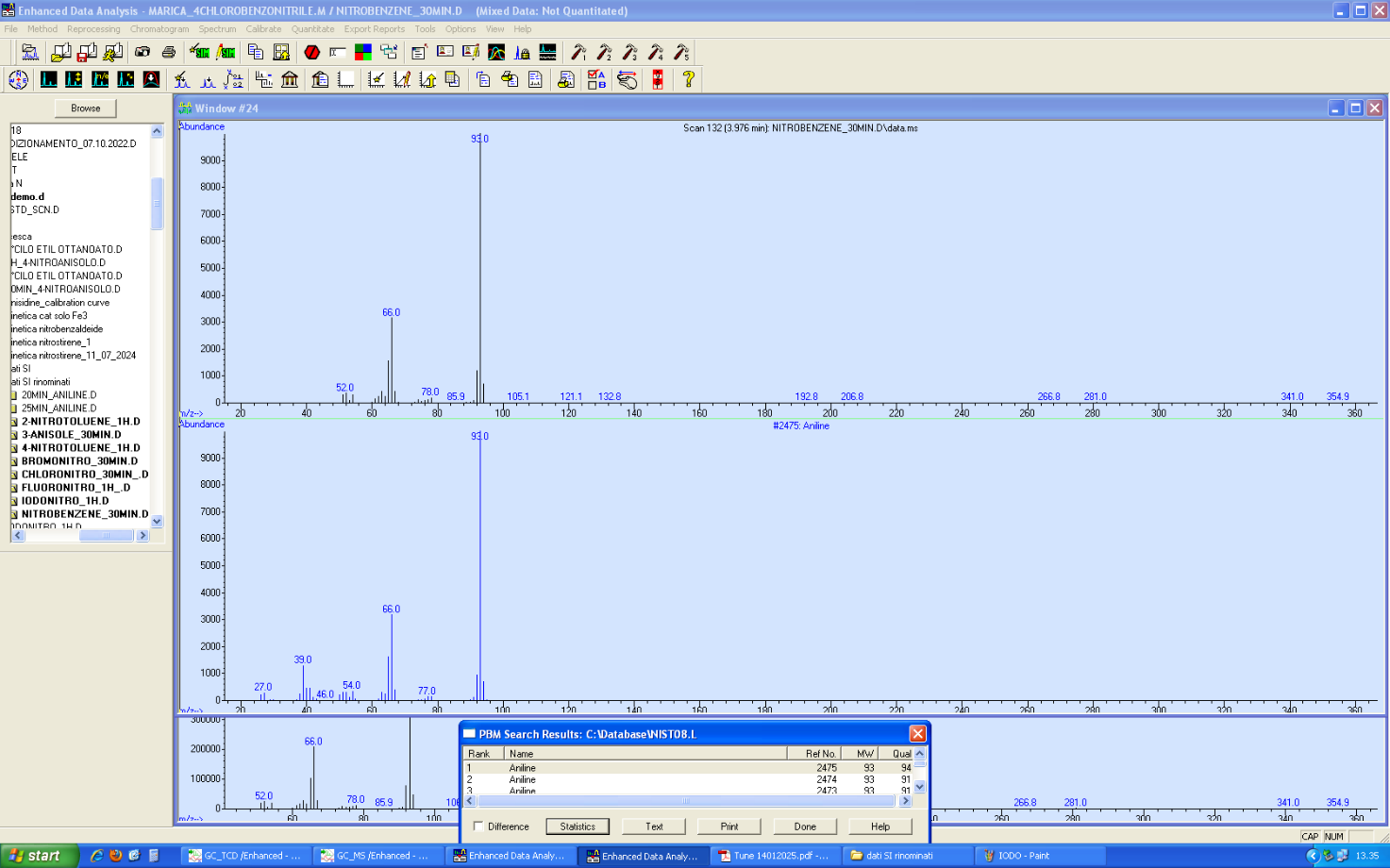


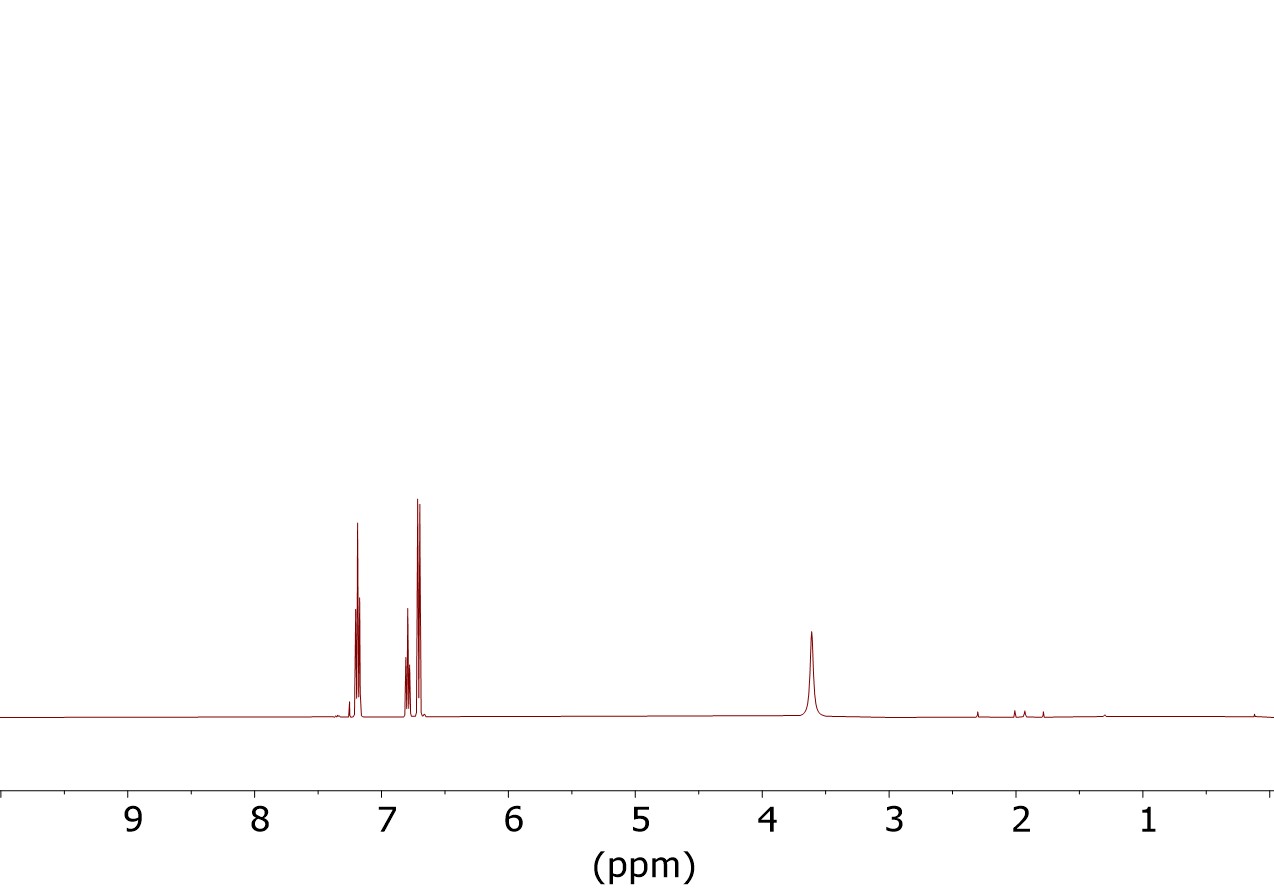
**Figure S3.** MS spectrogram of the obtained aniline. The comparison was made using NIST 0.8 Database.

**Figure S4.** ^1^H NMR spectrum of aniline obtained from scale-up experiment (CDCl_3_, 298 K, 500 MHz)


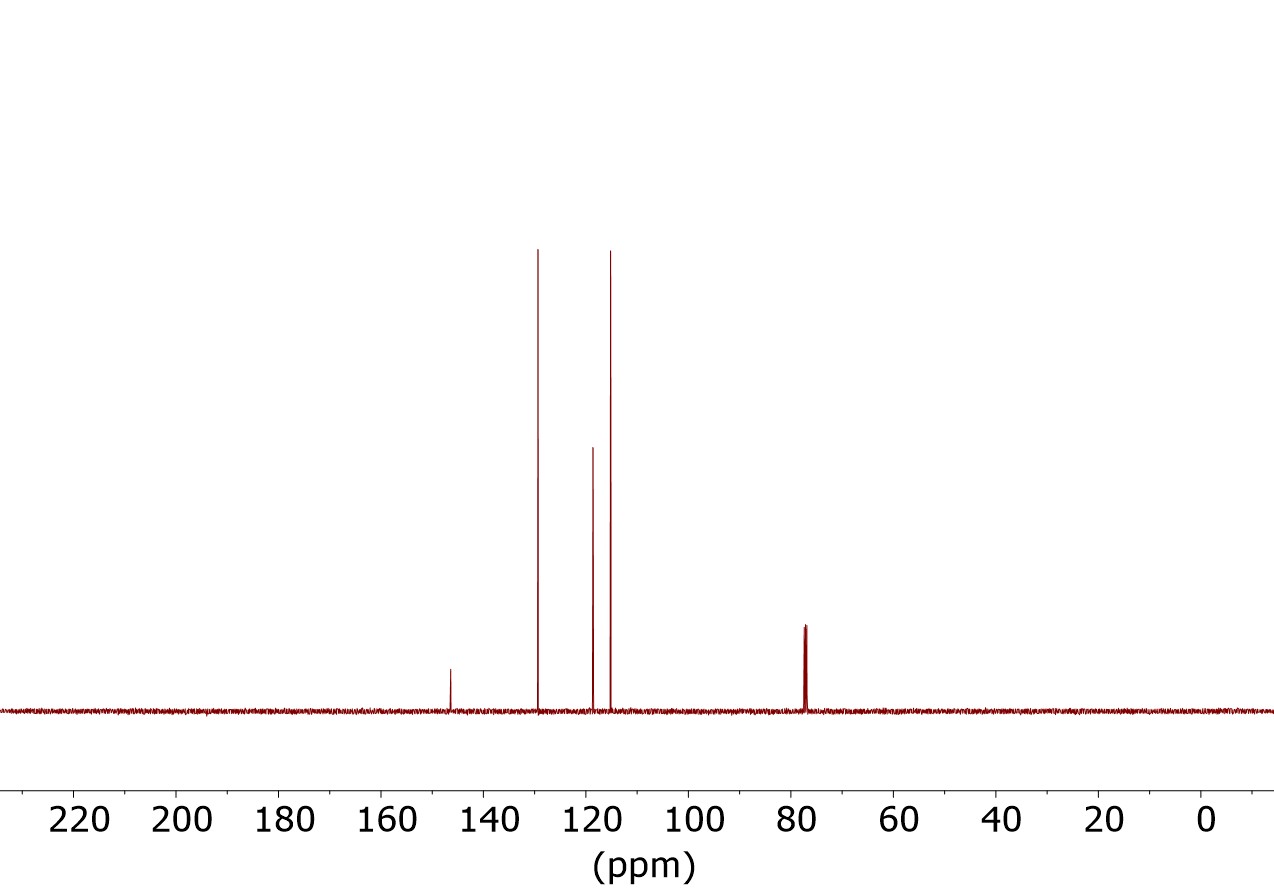


**Figure S5.** ^13^C{^1^H} NMR spectrum of aniline obtained from scale-up experiment (CDCl_3_, 298 K, 125 MHz)

S1.2 4-Fluoroaniline

Five different solutions were prepared using commercial 4-fluoroaniline and biphenyl in 5 mL of ethanol. Also in this case, the method employed at GLC consisted in an isothermal at 70 ºC for 30 sec, followed by an increase of 8 ºC/min until 280 ºC and an isothermal at this temperature for 5 min.

**Figure S6.** Calibration curve for the quantification of 4-fluoroaniline.

**Figure S7.** GLC plot of the sample retrieved from the reaction mixture after 60 min of reaction in which **Fe3** was used as catalyst.


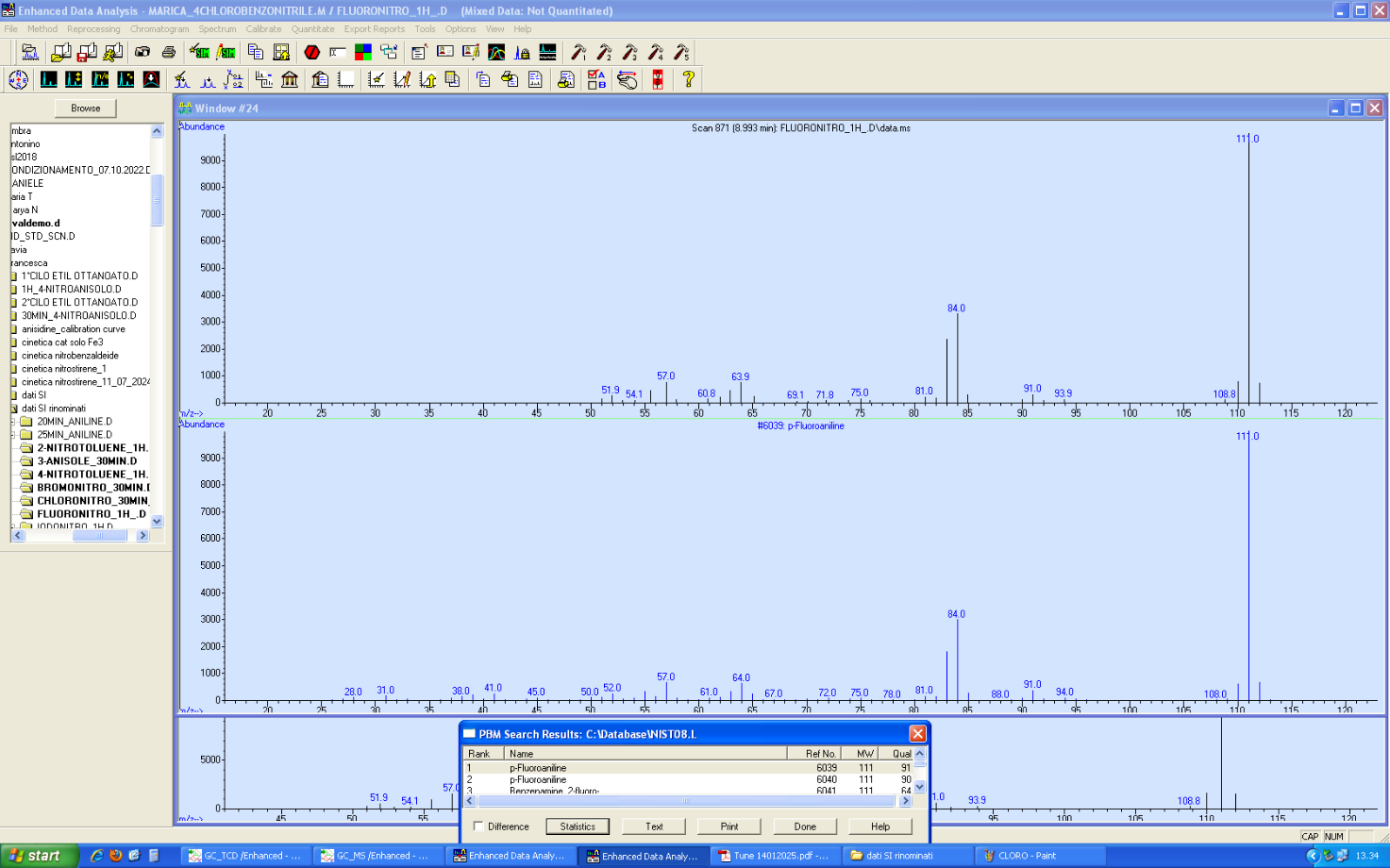


**Figure S8.** MS spectrogram of the obtained 4-fluoroaniline. The comparison was made using NIST 0.8 Database.

S1.3 4-Chloroaniline

Five different solutions were prepared using commercial 4-chloroaniline and biphenyl in 5 mL of ethanol. Also in this case, the method employed at GLC consisted in an isothermal at 70 ºC for 30 sec, followed by an increase of 8 ºC/min until 280 ºC and an isothermal at this temperature for 5 min.

**Figure S9.** Calibration curve for the quantification of 4-chloroaniline.

**Figure S10.** GLC plot of the sample retrieved from the reaction mixture after 30 min of reaction in which **Fe3** was used as catalyst.


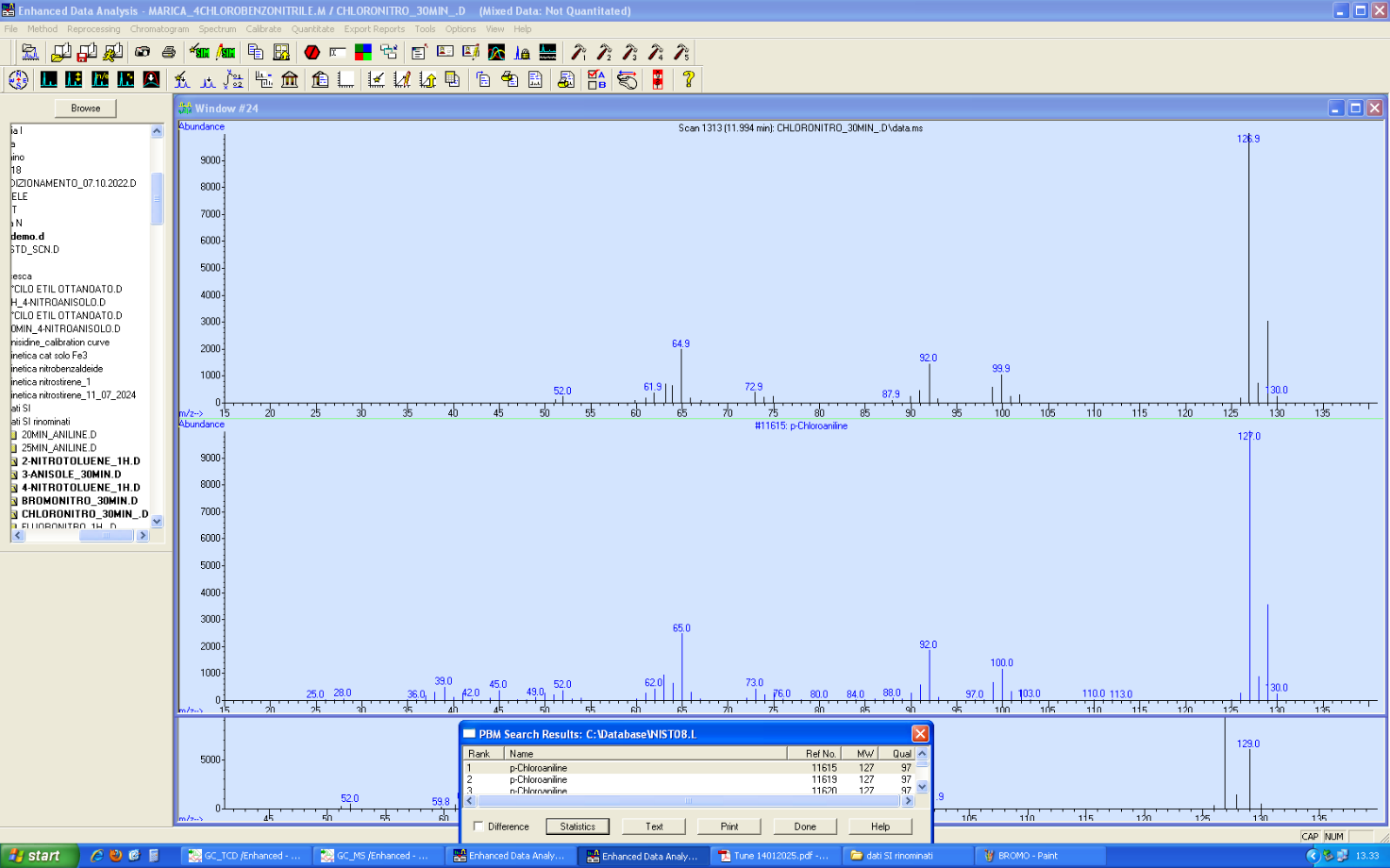


**Figure S11.** MS spectrogram of the obtained 4-chloroaniline. The comparison was made using NIST 0.8 Database.

S1.4 4-Bromoaniline

Five different solutions were prepared using commercial 4-bromoaniline and biphenyl in 5 mL of ethanol. In this case the method employed at GLC consisted in an isothermal at 130 ºC for 30 sec, followed by an increase of 5 ºC/min until 280 ºC and an isothermal at this temperature for 10 min.

**Figure S12.** Calibration curve for the quantification of 4-bromoaniline.

**Figure S13.** GLC plot of the sample retrieved from the reaction mixture after 30 min of reaction in which **Fe3** was used as catalyst.


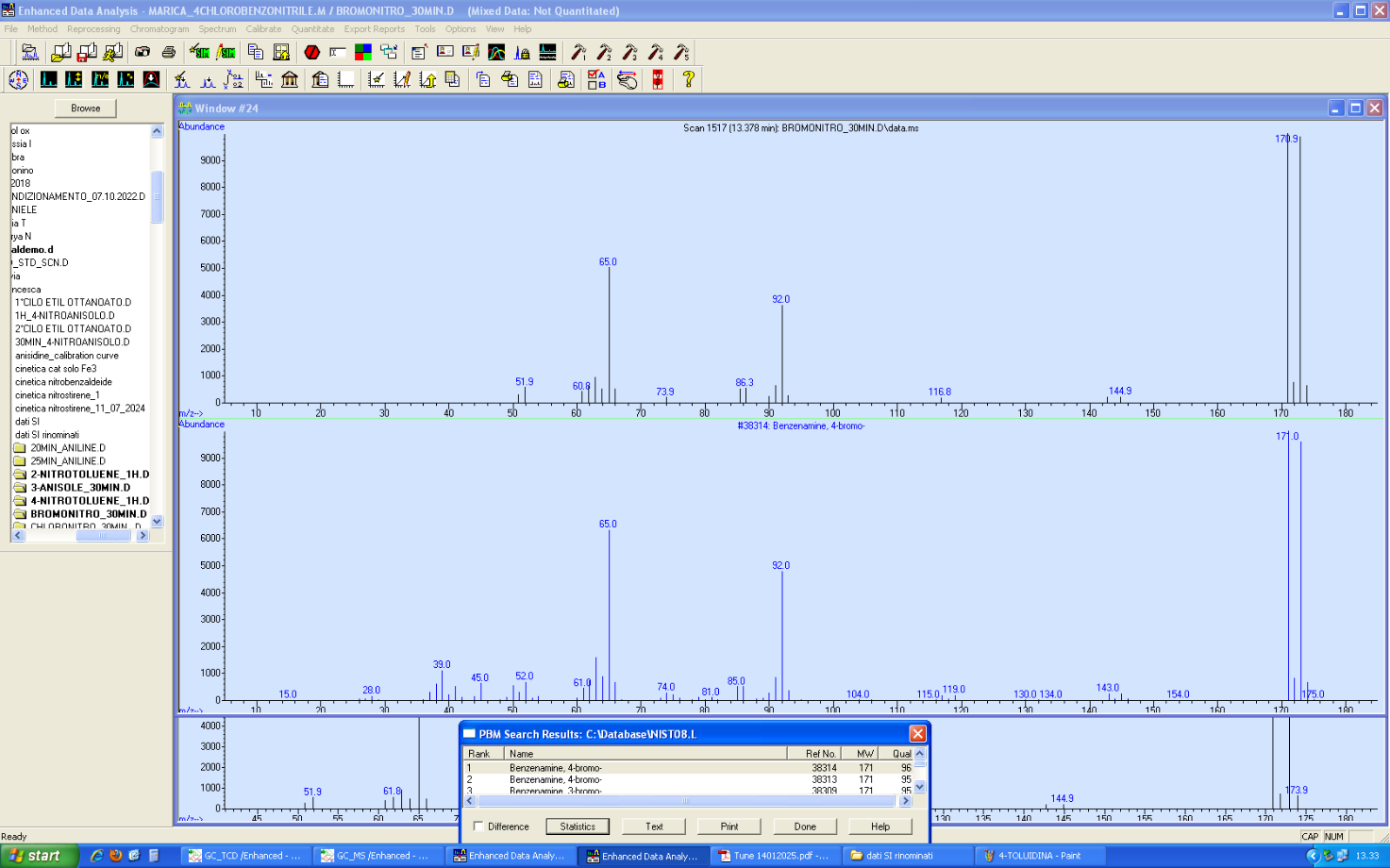


**Figure S14.** MS spectrogram of the obtained 4-bromoaniline. The comparison was made using NIST 0.8 Database.

S1.5 4-Iodoaniline

Five different solutions were prepared using commercial 4-bromoaniline and *n*-decane (internal standard) in 5 mL of ethanol. In this case the method employed at GLC consisted in an isothermal at 130 ºC for 30 sec, followed by an increase of 10 ºC/min until 280 ºC and an isothermal at this temperature for 10 min.

**Figure S15.** Calibration curve for the quantification of 4-iodoaniline.

**Figure S16.** GLC plot of the sample retrieved from the reaction mixture after 60 min of reaction in which **Fe3** was used as catalyst.


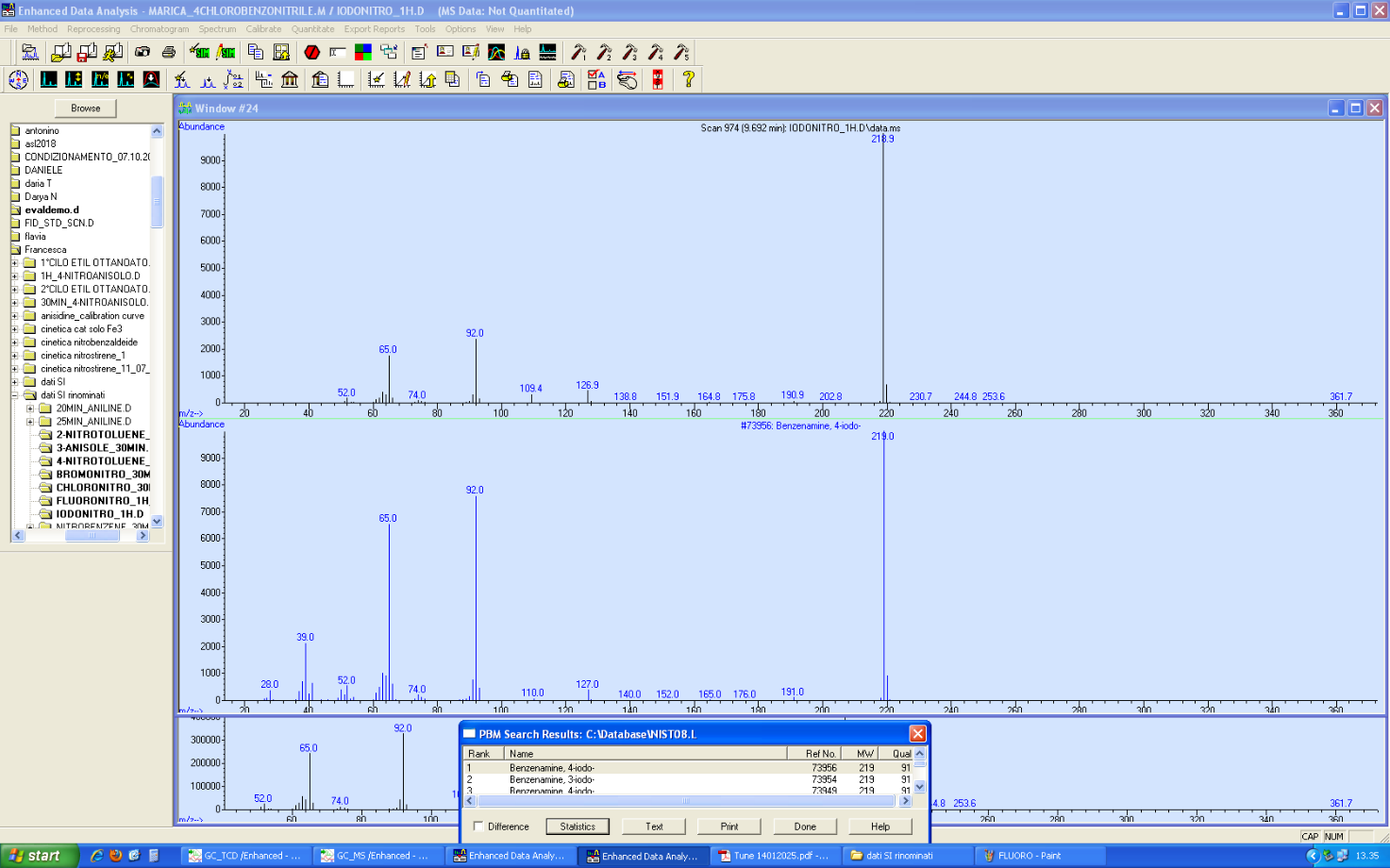


**Figure S17.** MS spectrogram of the obtained 4-iodoaniline. The comparison was made using NIST 0.8 Database.

S1.6 2-, 3-, 4-Toluidines

Five different solutions were prepared using commercial 4-toluidine and biphenyl in 5 mL of ethanol. The method employed at GLC consisted in an isothermal at 70 ºC for 30 sec, followed by an increase of 8 ºC/min until 280 ºC and an isothermal at this temperature for 5 min.

**Figure S18.** Calibration curve for the quantification of toluidines.

**Figure S19.** GLC plot of the sample retrieved from the reaction mixture after 60 min of reaction in which **Fe3** was used as catalyst.


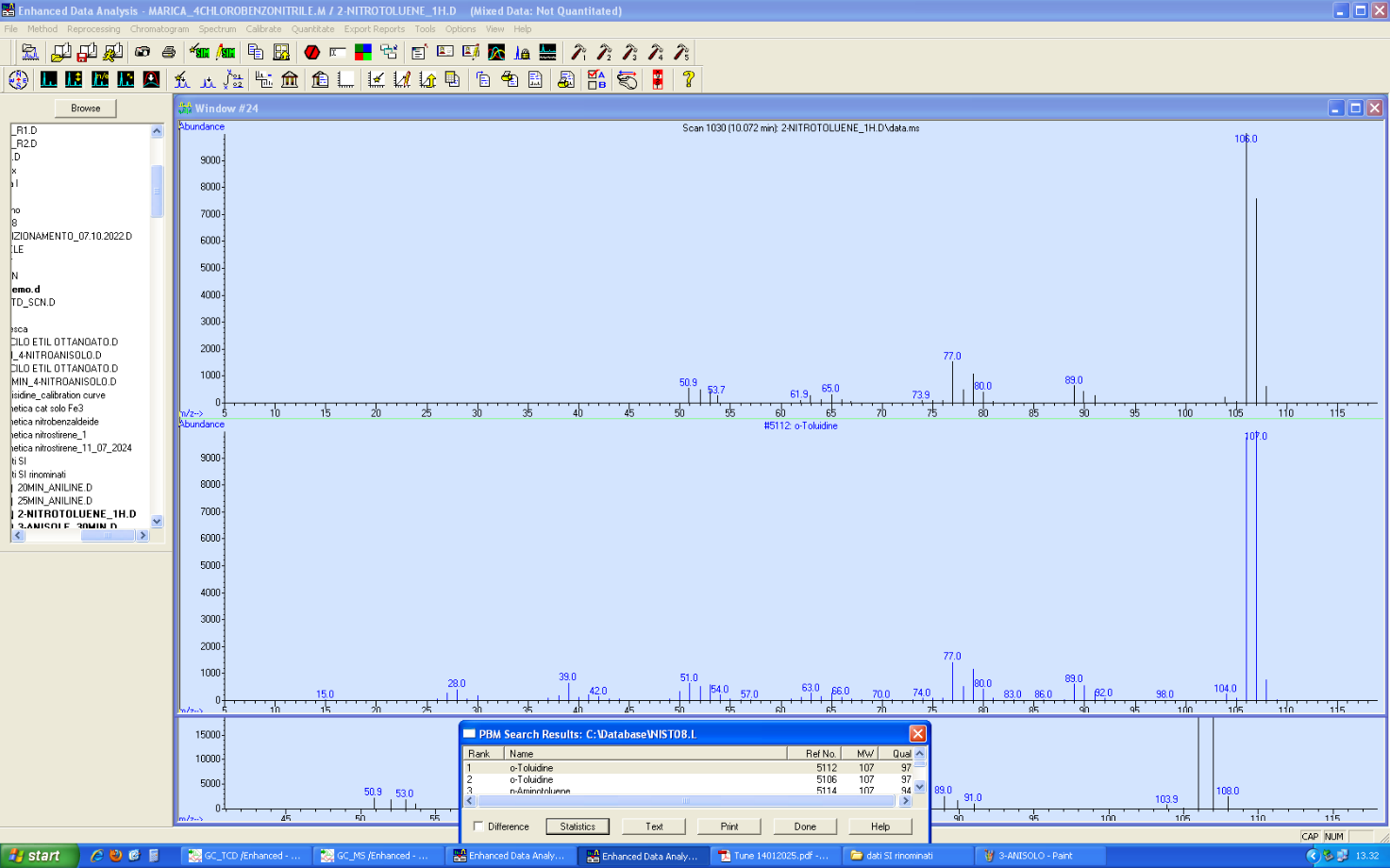


**Figure S20.** MS spectrogram of the obtained 2-toluidine. The comparison was made using NIST 0.8 Database.

**Figure S21.** GLC plot of the sample retrieved from the reaction mixture after 30 min of reaction in which **Fe3** was used as catalyst.


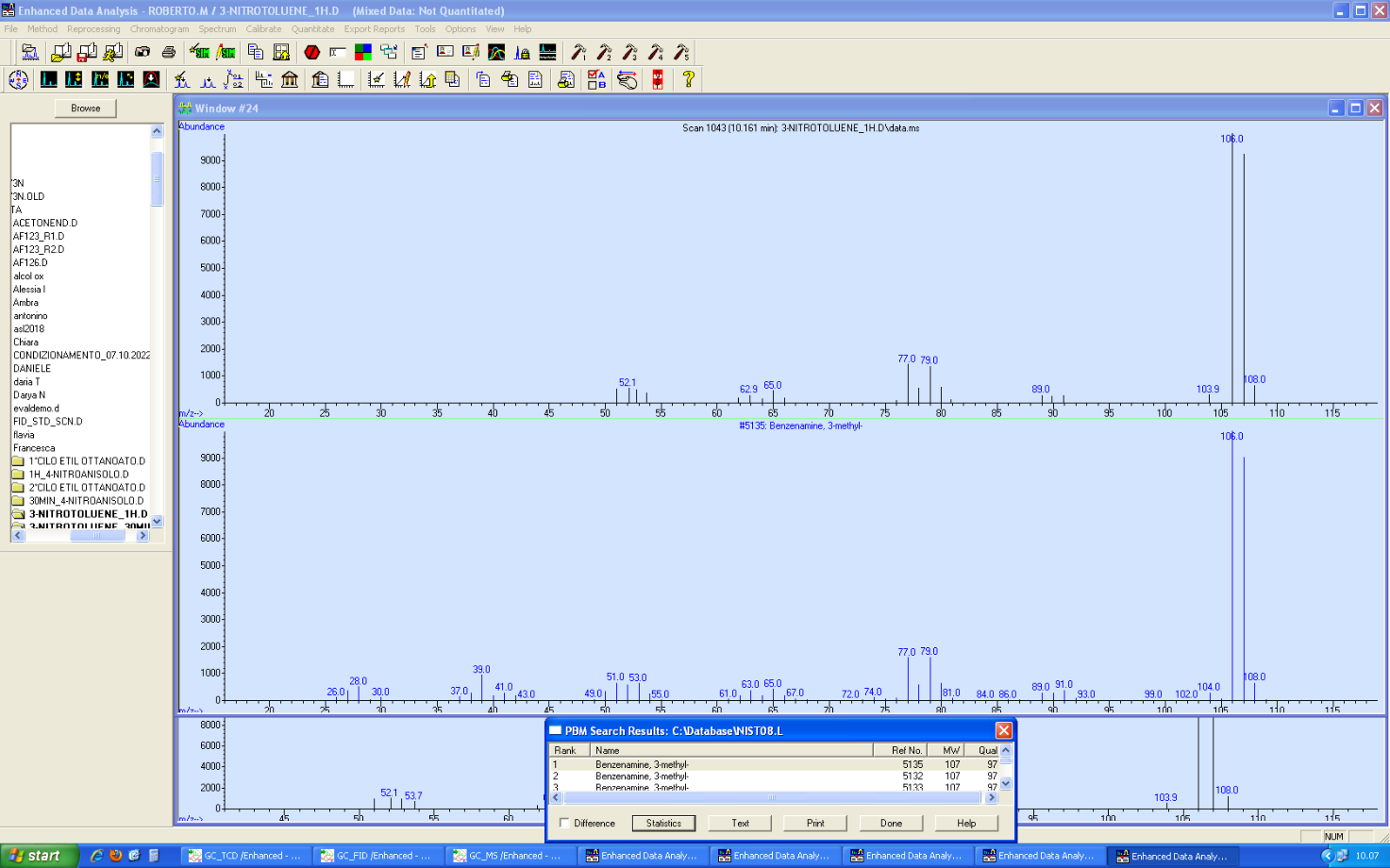


**Figure S22.** MS spectrogram of the obtained 3-toluidine. The comparison was made using NIST 0.8 Database.

**Figure S23.** GLC plot of the sample retrieved from the reaction mixture after 60 min of reaction in which **Fe3** was used as catalyst.


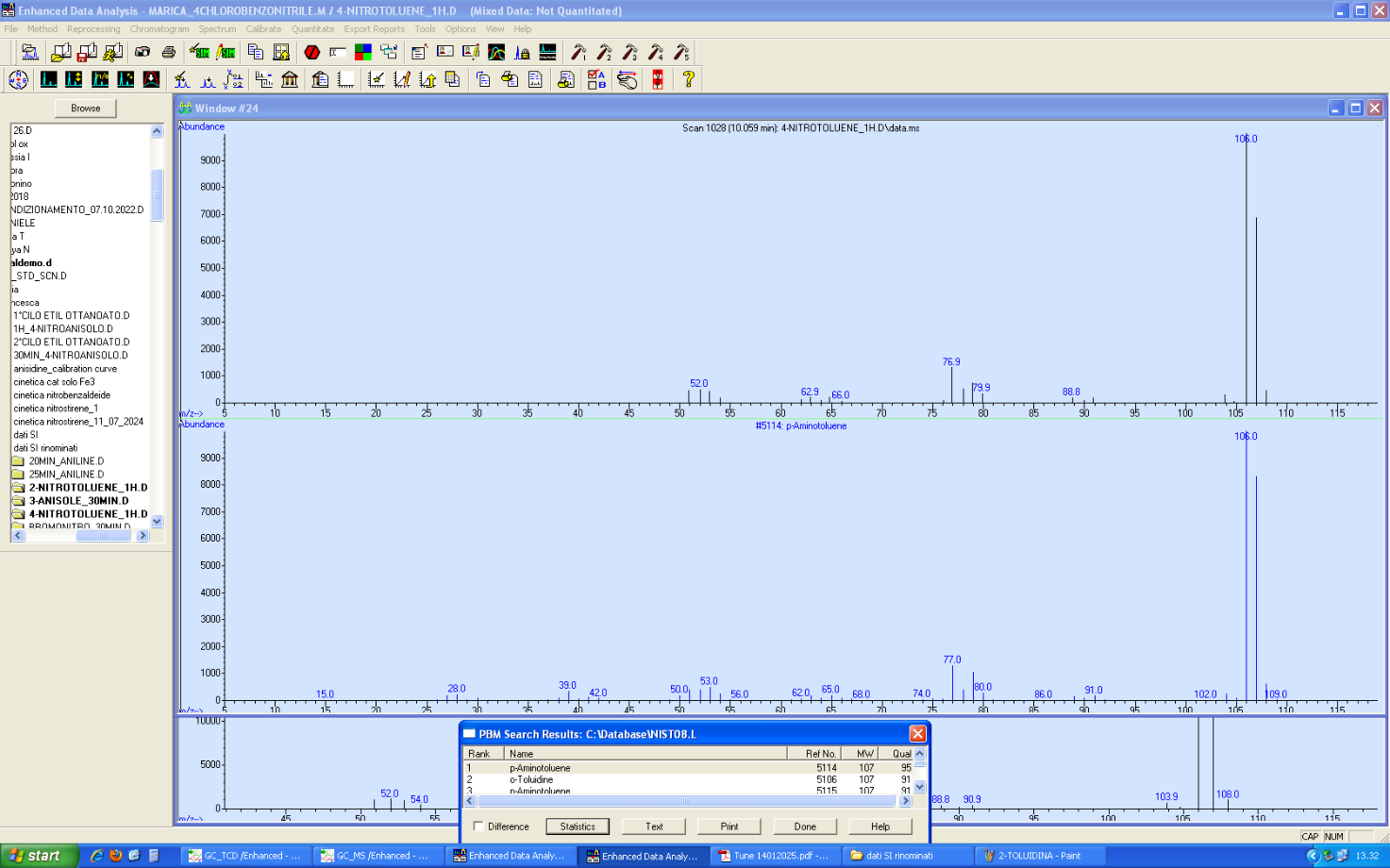


**Figure S24.** MS spectrogram of the obtained 4-toluidine. The comparison was made using NIST 0.8 Database.

S1.7 2-, 3-Anisidines

Five different solutions were prepared using commercial 2-anisidine and biphenyl in 5 mL of ethanol. The method employed at GLC consisted in an isothermal at 70 ºC for 30 sec, followed by an increase of 8 ºC/min until 280 ºC and an isothermal at this temperature for 5 min.

**Figure S25.** Calibration curve for the quantification of anisidines.

**Figure S26.** GLC plot of the sample retrieved from the reaction mixture after 30 min of reaction in which **Fe3** was used as catalyst.


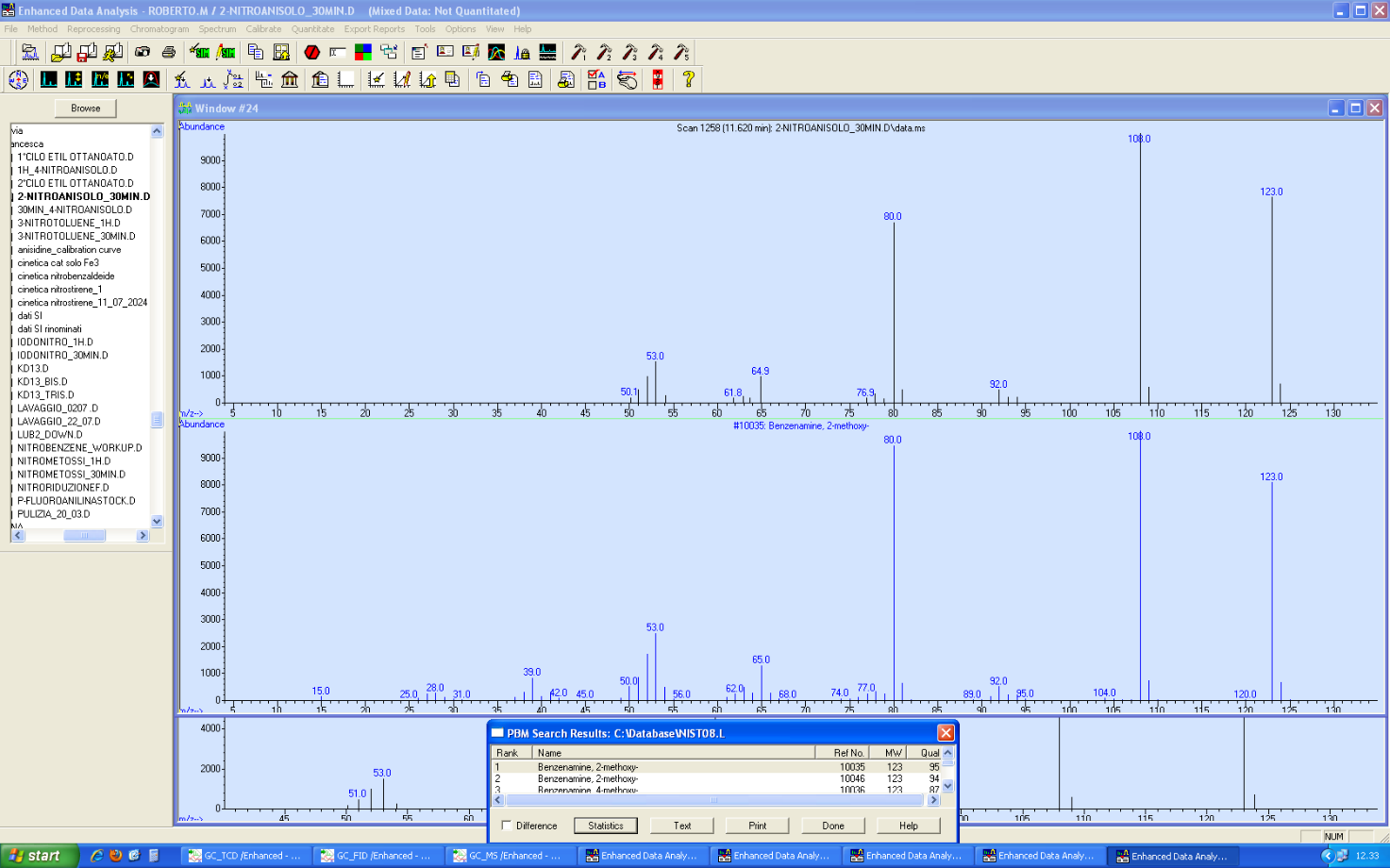


**Figure S27.** MS spectrogram of the obtained 2-anisidine. The comparison was made using NIST 0.8 Database.

**Figure S28.** GLC plot of the sample retrieved from the reaction mixture after 30 min of reaction in which **Fe3** was used as catalyst.


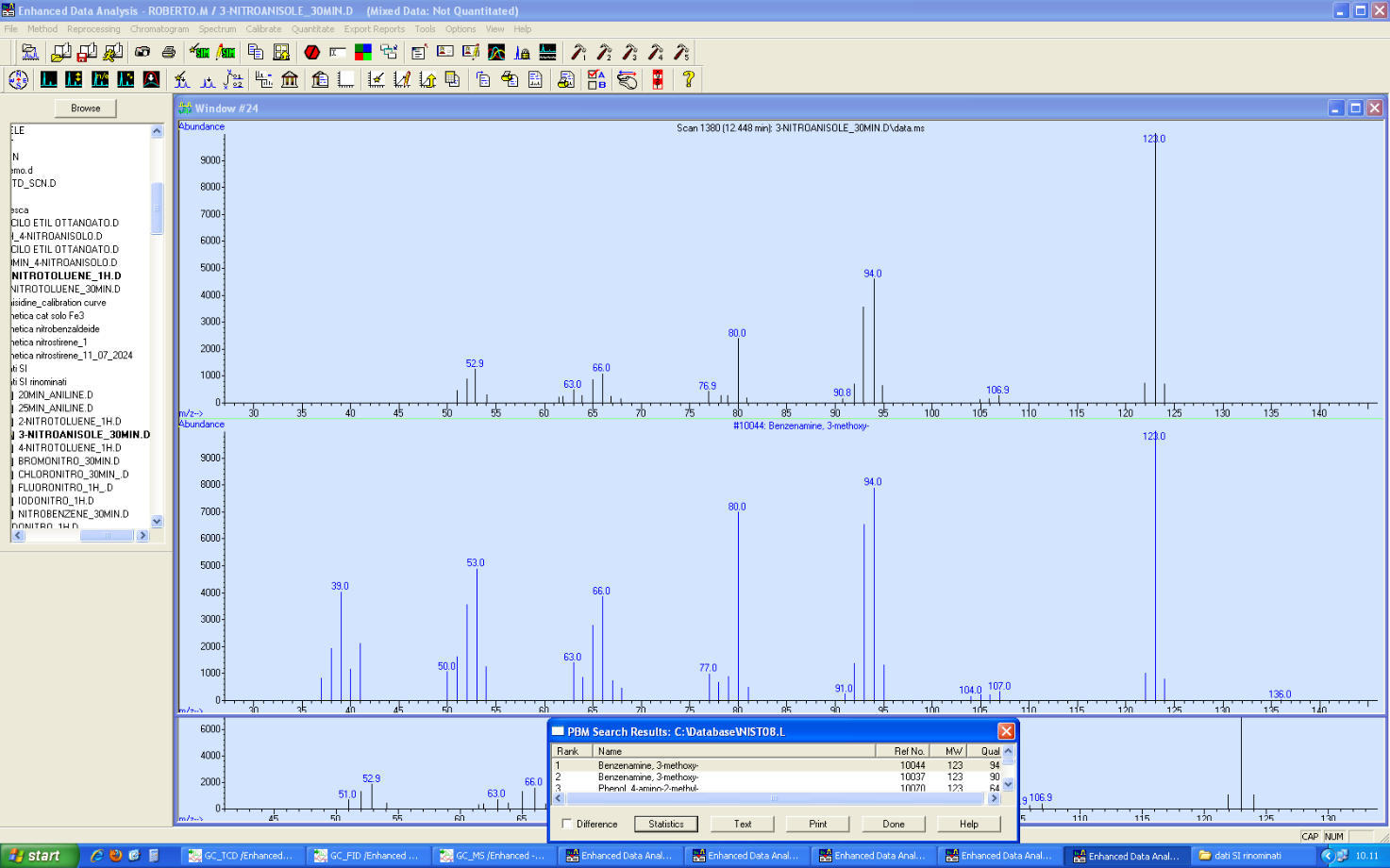


**Figure S29.** MS spectrogram of the obtained 3-anisidine. The comparison was made using NIST 0.8 Database.

S1.8 4-Aminophenol

Ten different solutions were prepared using commercial 4-nitrophenol and 4-aminophenol in 5 mL of ethanol. The obtained calibration curves are displayed in Figure S30 (calibration curve of 4-nitrophenol) and Figure S31 (calibration curve of 4-aminophenol). In both cases, the absorbance (at 402.5 nm for 4-nitrophenol, or at 314 nm for 4-aminophenol), was plotted against the molarity of the corresponding calibration solution.

**Figure S30.** Calibration curve for the quantification of 4-nitrophenol.

**Figure S31.** Calibration curve for the quantification of 4-aminophenol.

**Figure S32.** UV-Vis spectra obtained analysing the reaction mixture after 30 min and 60 min of reaction time (t_r_), when **Fe3** was employed as catalyst.

S2. Calculation of the corrected surface area

The corrected surface areas reported in Table 9 were calculated by the formula:

$$corrected surface area of sample= \frac{BET surface area of sample-BET surface area of SS}{fraction of ferrihydrite in the catalyst}$$

The fraction of ferrihydrite in the sample was calculated dividing the theoretical mass of ferrihydrite present in each catalyst (obtained assuming that all iron added during the preparation of the catalyst is converted into ferrihydrite) by the total mass of the synthesized catalyst.

**Table S1** Data used for the calculation of corrected surface areas.

| catalyst | precursor iron salt | mass of precursor iron salt  (g) | theoretical mass of ferrihydrite  (g) | total mass of the synthesized catalyst  (g) | fraction of ferrihydrite in the catalyst | surface area of SS  (m^2^/g) | BET surface area  (m^2^/g) | corrected surface area  (m^2^/g) |
| --- | --- | --- | --- | --- | --- | --- | --- | --- |
| **Fe3** | FeCl_3_·6H_2_O | 2.075 | 0.681 | 1.9893 | 0.342 | 2.18 | 179 | **517** |
| **Fe2** | FeSO_4_·7H_2_O | 2.1330 | “ | 2.9679 | 0.229 | “ | 63.6 | **268** |
| **Fe2_Cl** | FeCl_2_·4H_2_O | 1.523 | “ | 2.2323 | 0.305 | “ | 57.3 | **181** |

**Table S2** Elemental analysis (weight %) of **Fe3**, before and after duty, carried out by XRF analysis.

| Element | Pristine **Fe3** | **Fe3** after duty |
| --- | --- | --- |
| Fe | 39.2±0.7 | 40.5±0.3 |
| Ca | 9.0±0.10 | 7.9±0.2 |
| S | <LOQ | <LOQ |
| Si | 3.8±0.2 | 3.6±0.1 |
| Cr | 0.10±0.01 | 0.11±0.01 |
| Mn | 4.4±0.3 | 3.8±0.4 |
| Balance | 42.8±1.1 | 41.7±0.9 |

S3. Supplementary characterisation results

S3.1 FTIR spectra of catalyst Fe3 before and after duty

**Figure S33** FTIR-ATR spectra of catalyst **Fe3** before (brown line) and after (pink line) duty. The upper spectrum (black line) is that of pristine **SS**. The absorption bands at ca. 3320 cm^−1^ and 1645 cm^−1^ can be attributed to OH groups of ferrihydrite,^[97]^ while the band at 1450 is assigned to CO_3_^2–^ stretching.

S3.2 X-Ray diffraction pattern of Fe2_Cl

**Figure S34** X-Ray diffraction pattern of catalyst **Fe2_Cl**

S3.3 XP spectra


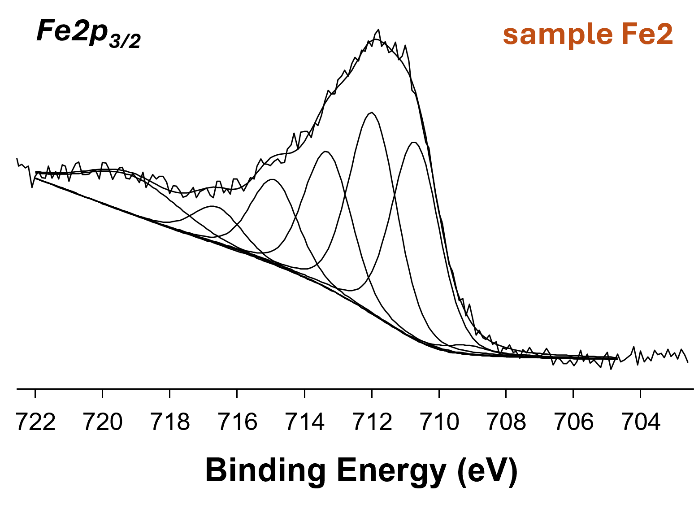


**Figure S35** Curve fitted Fe2p_3/2_ XP spectrum of **Fe2**.


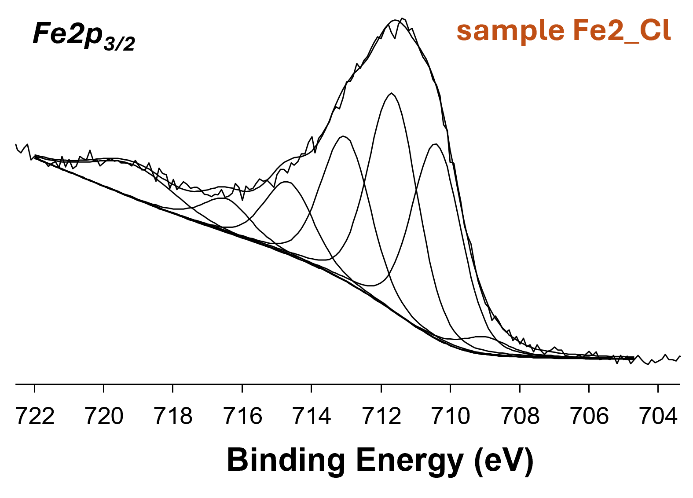
**Figure S36** Curve fitted Fe2p_3/2_ XP spectrum of **Fe2_Cl**.


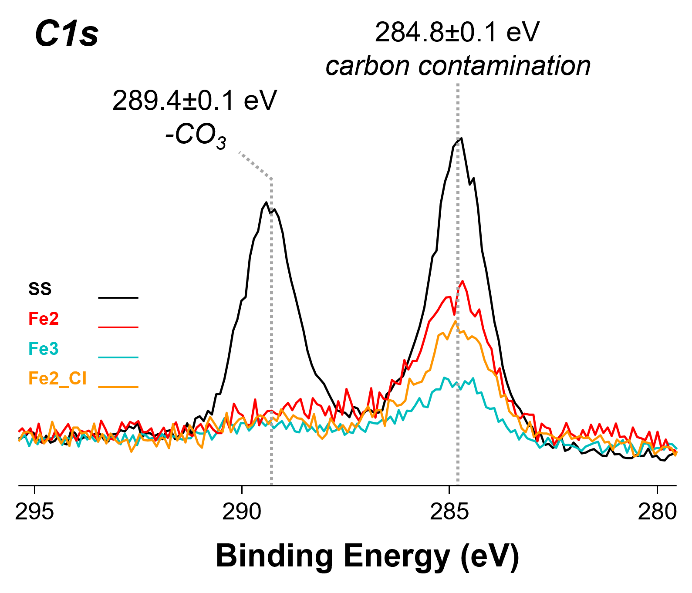


**Figure S37** C1s XP spectra of **SS**, **Fe2**, **Fe3**, and **Fe2_Cl**.


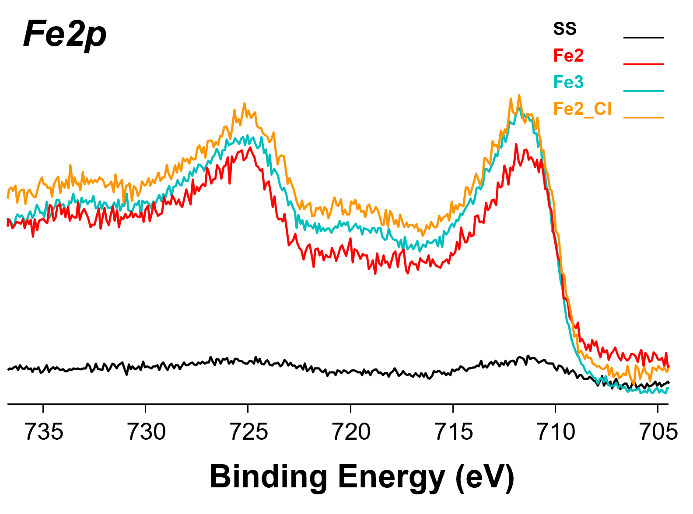


**Figure S38** Fe2p XP spectra of **SS**, **Fe2**, **Fe3**, and **Fe2_Cl**.


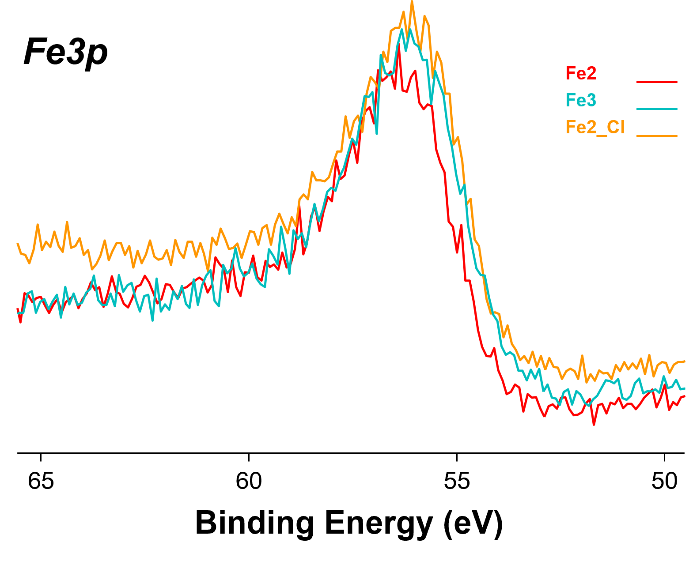


**Figure S39** Fe3p XP spectra of **Fe2**, **Fe3**, and **Fe2_Cl**.

S3.4 Images of catalysts suspensions obtained after sonication


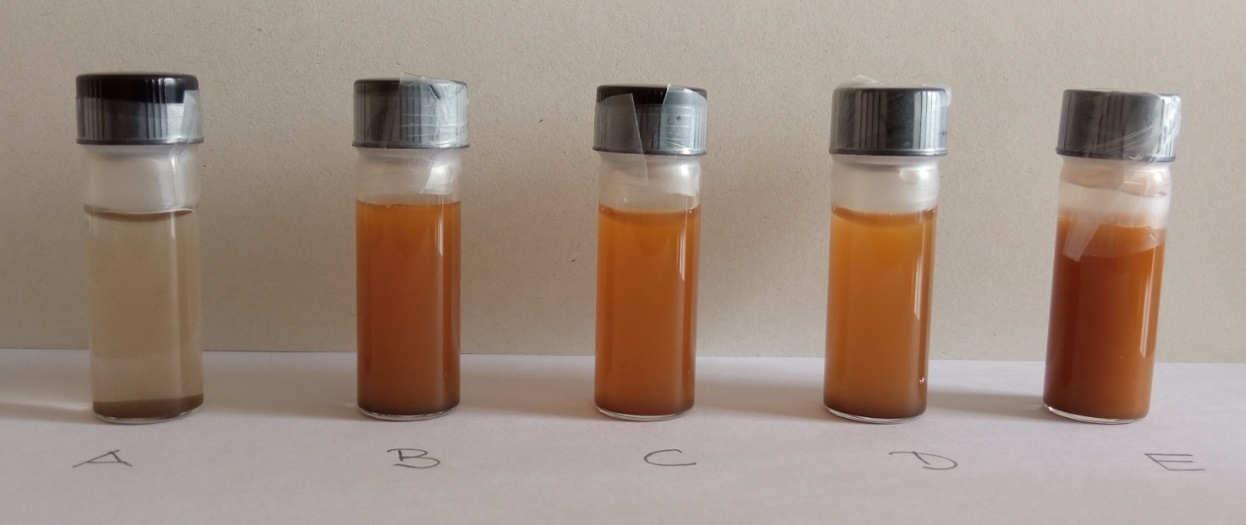


**Figure S40** Aqueous suspension obtained after sonication at room temperature for 20 minutes: (A) as-received steel slag (**SS**), (B) **Fe3** fresh catalyst, (C) **Fe2** fresh catalyst and (D) **Fe3** catalyst after one catalytic run.


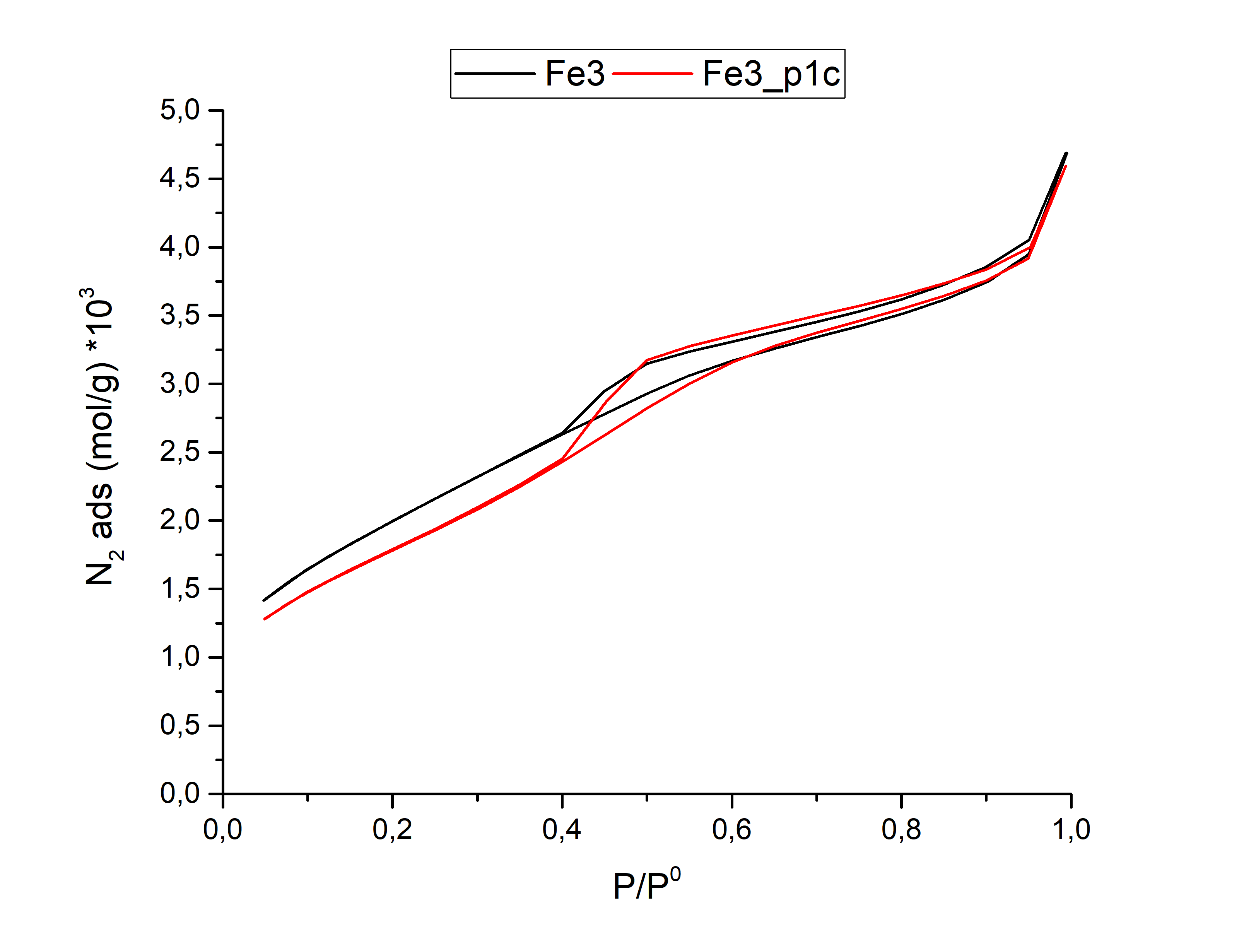
S3.5 Nitrogen physisorption isotherms of Fe3 before and after one catalytic run

**Figure S41** Nitrogen physisorption isotherms of **Fe3** before (black line) and after one catalytic run (red line).

**Reference**

[97] J. D. Russell, “Infrared spectroscopy of ferrihydrite: evidence for the presence of structural hydroxyl groups” *Clay Minerals*. **1979**;*14*, 109-114.
